# Processing of collagen based biomaterials and the resulting materials properties

**DOI:** 10.1186/s12938-019-0647-0

**Published:** 2019-03-18

**Authors:** Michael Meyer

**Affiliations:** Research Institute for Leather and Plastic Sheeting, Meissner Ring 1-5, 09599 Freiberg, Germany

## Abstract

Collagen, the most abundant extracellular matrix protein in animal kingdom belongs to a family of fibrous proteins, which transfer load in tissues and which provide a highly biocompatible environment for cells. This high biocompatibility makes collagen a perfect biomaterial for implantable medical products and scaffolds for in vitro testing systems. To manufacture collagen based solutions, porous sponges, membranes and threads for surgical and dental purposes or cell culture matrices, collagen rich tissues as skin and tendon of mammals are intensively processed by physical and chemical means. Other tissues such as pericardium and intestine are more gently decellularized while maintaining their complex collagenous architectures. Tissue processing technologies are organized as a series of steps, which are combined in different ways to manufacture structurally versatile materials with varying properties in strength, stability against temperature and enzymatic degradation and cellular response. Complex structures are achieved by combined technologies. Different drying techniques are performed with sterilisation steps and the preparation of porous structures simultaneously. Chemical crosslinking is combined with casting steps as spinning, moulding or additive manufacturing techniques. Important progress is expected by using collagen based bio-inks, which can be formed into 3D structures and combined with live cells. This review will give an overview of the technological principles of processing collagen rich tissues down to collagen hydrolysates and the methods to rebuild differently shaped products. The effects of the processing steps on the final materials properties are discussed especially with regard to the thermal and the physical properties and the susceptibility to enzymatic degradation. These properties are key features for biological and clinical application, handling and metabolization.

## Introduction

More than 2000 years ago early surgeons already used collagen-based materials as skin or intestine to close wounds and for reconstructive surgery. However, only the past 50 years brought a more frequent use of collagen as medical product because technologies of intensive cleaning and sterilization procedures were developed [1]. Applications among others are wound closure, treatment of burns, hemostasis, hernia repair, repair of bone and cartilage defects, as well as various dental applications including guided bone repair [2–4].

In tissues collagen is the scaffold material which provides an optimal environment for physiologically highly active cells and cellular components. Therefore, recent developments focus on the decellularization of organ parts or whole organs, maintaining the tissue architecture followed by recellularization to overcome the high need of organs for organ transplantation. Important progress is also expected by using collagen-based bioinks which can be combined with live cells and which are formed into 3D structures.

During the last decades many reviews summarized the different applications of collagen as biomaterial because of this important function of collagen as structure forming material [2, 3, 5–9]. It is used as drug delivery system [4, 10, 11], as matrices for tissue engineering [12, 13], topical hemostyptics [14], for soft tissue repair [15], and as membrane for diverse applications [16, 17]. Recent contributions reviewed technological aspects but mainly for organ and tissue decellularization [18–25].

This review will give an overview about the principles of processing collagen-rich tissues down to collagen hydrolysates, the rebuilding of differently shaped materials and the effects of the processing steps on the final materials properties especially the thermal and the physical properties and the susceptibility to enzymatic degradation. These properties are key features for clinical handling and degradation behaviour. This is all the more important with regard to the technological progress in organ decellularization, that aims to save structures and additive manufacturing, where new structures are rebuilt from smaller units [26].

Collagen processing technologies are organized as a series of steps. Several steps are combined in different ways to manufacture structurally versatile materials with varying properties with regard to shape, mechanics, physiological behaviour and handling. Processing may either maintain the collagen structure or affect it by intensive chemical, mechanical and physical treatment. Reassembly of fibres and shaping allows to generate new structures. The different processing steps to treat tissues are summarized to discuss the effects of these steps on the final properties and to serve as a playground for a plethora of different ECM derived structures which can be used in medical and pharmaceutical applications in solid or liquid form as well as to manufacture 2D or 3D structures.

## Collagen raw materials and sources for medical uses

Many collagen-rich tissues are used as raw materials to manufacture medical products for surgical purposes e.g. as soft tissue augmentation to support wound healing, in dental applications and other applications. Typically skin, pericard, small intestine, urinary bladder and tendon are applied [27] but also many other tissues as bone, fasica lata, heart valves etc. Beside tendon and bone all of these tissues can be described as stratified compositions of fibrous proteins, associated with different non-fibrous substances, cells and cellular components. According to their function the tissues are composed of layers. These layers can take over mechanical forces or they act as highly metabolic part of the tissue with high cell load. The metabolic layers show membraneous activity responsible to separate or transport chemical molecules or cellular components.

Biomedical companies manufacture implants from those tissues from human or animal sources. The tissues are purified and often processed in many ways. Table 1 summarizes selected products differing in structure, crosslinking technology, tissue source and species and their sterilization technique. More comprehensive collections are published by other authors [27–31]. Recent investigations described principles to decellularize organs and organ parts, which shall be recellularized, but such products have not yet come into the market. In the following sections histological images of the most common tissues, which require a more intensive technological treatment to prepare medical products, are described as well as the parts of the tissues which are saved during the process.

**Table 1:** Selected marketed collagen products

Company	Product	Raw Material	Species	CL	Additive	Form		Sterilisation	Indication
Geistlich Biomaterials	Biogide	Dermis	Porcine	–	–	Membrane	2b	Gamma	Dental
Zimmerdental	Biomend	Tendon	Bovine	GA	–	Membrane	2	Ethylenoxide	Dental
Arthrothek	CuffPatch	SIS	Porcine	+	–	Hydrated sheet	1		Soft tissue augmentation
Synovis Surgical Baxter	Dura-Guard	Pericardium	Bovine	GA	–	Hydrated sheet	1	Aseptic prod	Spinal and cranial repaeir
DIZG	Epiflex	Dermis	Human	–	–	Membrane	1	Aseptic prod	Dermis replacement; soft tissue reconstruction
Gelita Medical	GelitaSpon	Gelatin	Bov or porc	FA	–	Sponge	3	Gamma	Hemostasis
Resorba	Gentacoll	Tendon	Equine	–	Antibiotic	Sponge		Ethylenoxide	Hemostasis, dental
Medskin solutions Dr. Suwelack	Matriderm	Dermis	Bovine	DHT	Elastin	Membrane	2	Gamma	Burns IIb–III; trauma, reconstructive and surgical wounds
Medskin solutions Dr. Suwelack	Matristypt	Dermis	Bovine	DHT	–	Membrane	2	Gamma	Hemostasis
MBP	MB-collagen	Dermis	Porcine	–	–	Membrane	2	Gamma	Burns IIb–III; trauma, reconstructive and surgical wounds
Cook Biotech	Oasis	SIS	Porcine	–	–	Membrane	1	Ethylenoxide	Burns IIb–III; trauma, reconstructive and surgical wounds
Resorba	Parasorb Cone	Tendon	Equine	–	–	Sponge	2	Ethylenoxide	Hemostasis, dental
Covidien	Permacol	Dermis	Porcine	HMDI	–	Membrane	1	Gamma	Soft tissue repair
Lifecell Corp.	Strattice	Dermis	Porcine	–	–	Membrane	1	eBeam	Hernia and abdominal wall repair
MBP	Surgicoll	Dermis	Porcine	–	–	Sponge	2	Gamma	Hemostasis
C.R. Bard	XenMatrix	Dermis	Porcine	–	Antibiotic	Membrane	1	eBeam	Soft tissue and hernia repair
MBP	Xenoderm	Dermis	Porcine	–	–	Membrane	1	Gamma	Soft tissue and hernia repair
MBP	Xenoguard	Pericardium	Porcine	–	–	Membrane	1	ETO	Soft tissue reconstruction
	Zyderm	Dermis	Bovine	–	–	Solution	3	Filtration	Soft tissue augmentation in plastic surgery

The products differ in structure, crosslinking degree and technology, tissue and species source, and its indication and additives [16, 28–31]

GA, glutaraldehyde; DHT, dehydrothermal treatment; HMDI, hexamethylendiisocyanate; FA, formaldehyde; 1, intact tissue structure; 2, suspension; 2b, combination of 1 and 2; 3, solution

### Skin

The skin of vertebrates encloses the whole body and shields it against environmental impact. For long time skin has been discussed as largest organ [32, 33] but neither by weight nor by area this seems to be true [34]. However, skin is an organ which can be easily transferred into final materials with large area.

**Fig. 1:**
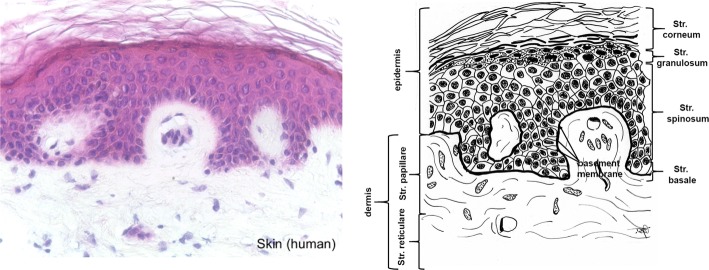
Histological cut (left) of human skin and corresponding drawing. Epidermal layers and the cellular components of the dermis are removed during processing, while saving the structural components of the dermis. (All histological pictures and drawings were kindly supplied by Gundula Schulze-Tanzil, Institut für Anatomie, Paracelsus Medizinische Privatuniversität (PMU), Salzburg and Nürnberg, Nürnberg, Germany)

In mammals the skin consists of the three layers epidermis, dermis and subcutis (Fig. 1). Thick, highly keratinized skin with 12–20 cell epidermal layers is found in the areas of the body with high tear (palms, soles), thin skin (2–4 cell layers) covers the rest of the body. The epidermis of thick skin is subdivided into four layers: stratum basale, stratum spinosum, stratum granulosum, and stratum corneum. The basal layer (stratum basale or germinativum) consists of mitotically active keratinocytes on a basement membrane which consists of collagen type IV adjacent to the dermis [35]. The other layers consist of cells in different states of transformation. As the most external layer the stratum corneum consists of cells without nucleus which are keratinized and which desquamate. The colour of the skin is caused by pigments produced by melanocytes originating from the neural crest, and which are found in the basal layer [32, 36].

The dermis is subclassed into papillary layer (Str. papillare) and reticulary layer (Str. reticulare). The papillary layer forms papillae at the dermal–epidermal junction zone. These papillae increase the surface of the junction zone and improve adhesion of the layers. Hair follicles extend into the papillary layer. They are faced with epidermis and contain the hair root with the bulb. Bulb cells proliferate to build the hair.

While the epidermis consists of the structure protein keratin, the dermis is mainly built from collagen fibres which partly co-organize with elastin fibers [37]. Furthermore, the fibrous tissue contains a mixture of diverse macromolecules (hyaluronic acid, dermatane sulfate, chondroitin sulfate, fibronectin, tenascin, epimorphin and others), which bind high amounts of water, filling the space between the fibres [33].

The subcutis contains a mixture of collagen fibres and associated components, especially elastin and fat tissue, which can be a layer beneath the skin in well nourished animals, but which can also be organized in form of papillae which provide an insight into the dermis (e.g. porcine skin). The collagen fibres of the subcutis interweave with the collagen fibres in the dermis. The skin also contains diverse glands (sweat, odour, sebaceous), muscles (e.g. musculus attractor pili), nerves, receptors and cells.

The fibre orientation in skin is in homogenous regarding the area, the cross-sections and between skins of different animals of the same species [38–40]. The skin shows an internal tension which is observed along the Langer lines (± 10°) and which is caused by a passive pretension of the collagen fibres. The most homogenous part with lowest tension is found in the central region which covers the loins. The ventral zones and the extremities show higher pre-stress and are much more elastic [41, 42]. Furthermore, the mechanical constitution of the skin depends on species and age and presumably other factors [40–42]. Purification procedures usually remove epidermis and subcutis. Depending on the thickness of the raw material the dermis can be split into a grain part, which contains the papillary layer and a part which consists mainly of the reticularis.

### Pericardium

**Fig. 2:**
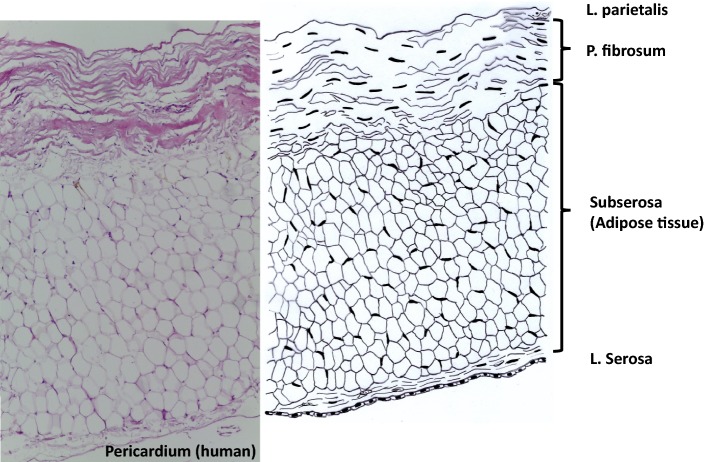
Histological cut (left) of human pericardium and corresponding drawing. During processing only the P. fibrosa is saved

The pericardium completely covers the heart in form of a sac. It consists of three layers and additionally surrounding adipose tissue and a serosa covering the latter (Fig. 2). The Pericardium fibrosum is responsible to prevent the heart from overexpansion and the P. serosum is further divided in two layers. The Lamina parietalis is tightly adhered to the P. fibrosum, the Lamina visceralis (not shown) is separated from the other lamina by a slippy liquid layer and covers the myocardium [43]. P. fibrosum and L. visceralis consist of collagen and elastic fibers [44]. The collagen fibers especially in the P. fibrosum are highly oriented. The mechanical stability is therefore not uniformly distributed, but the tissue behaves anisotropic [45]. Decellularization removes the serosae and adipose tissue [46].

### Intestine

**Fig. 3:**
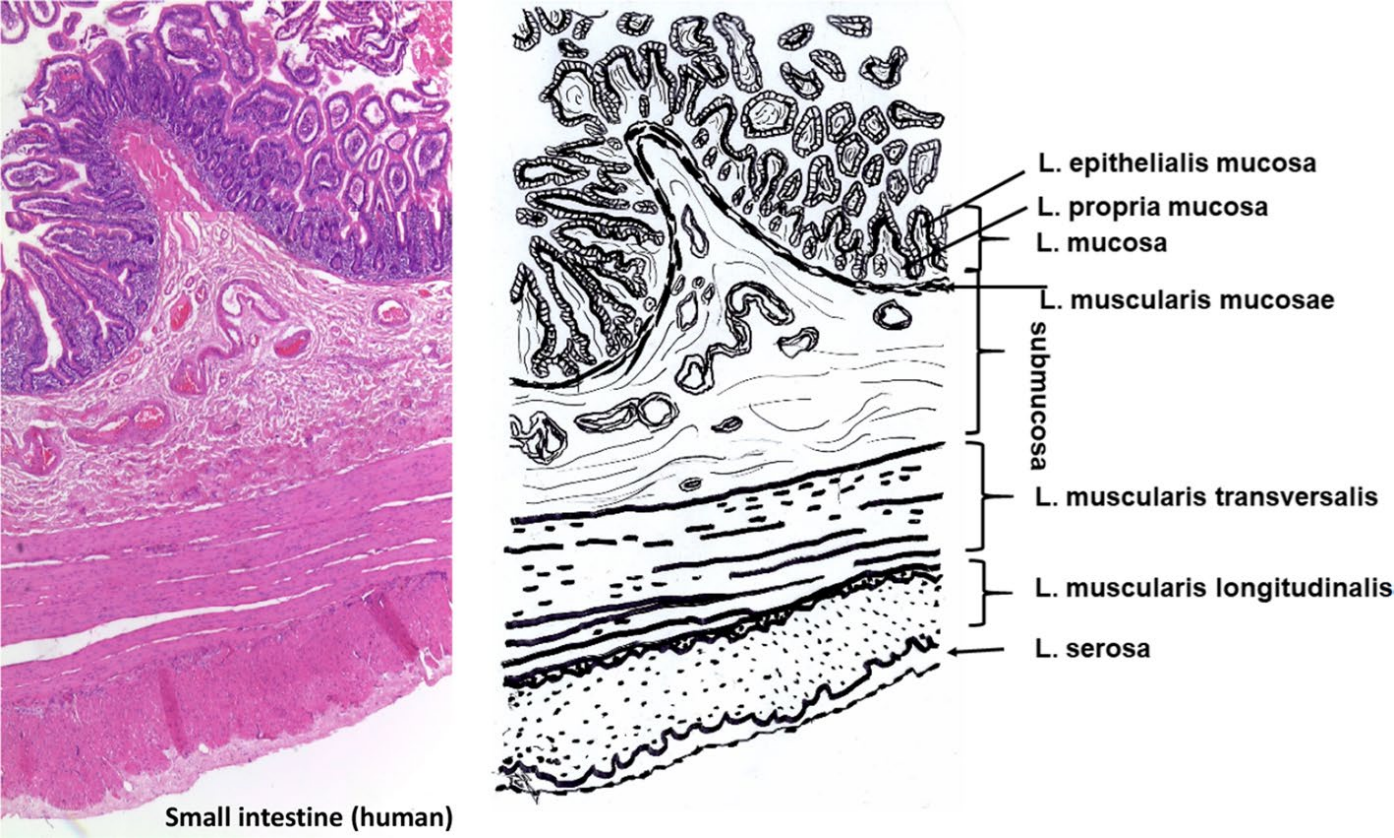
Histological cut (left) of human small intestine and corresponding drawing. Processing saves submucosa and parts of muscularis transversalis. Mucosa, muscularis longitudinalis and serosa are removed

Small intestine constists of five layers beginning with the lumen (Fig. 3): Laminae mucosae (3 laminae), submucosa, L. muscularis transversalis, L. muscularis longitudinalis and L. serosae [47]. During decellularization only the submucosa and parts of the muscularis transversalis persist. The other layers are completely removed.

### Urinary bladder

**Fig. 4:**
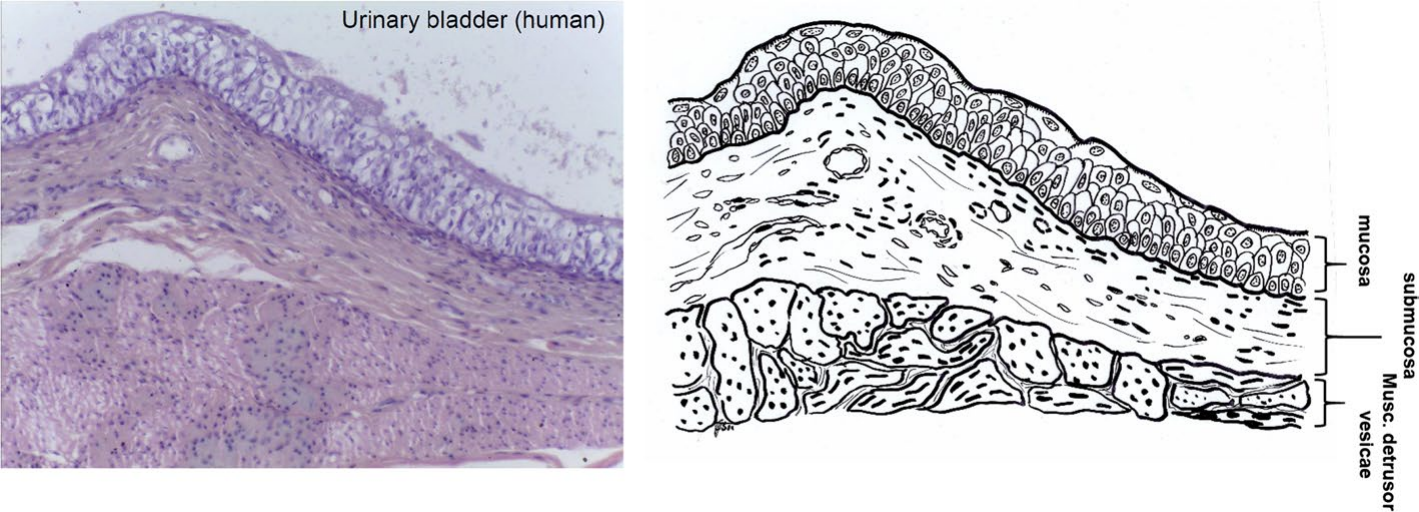
Histological cut (left) of human urinary bladder and corresponding drawing. Processing saves only the submucosa

Similar to intestine, the urinary bladder tissue is formed of Laminae mucosae, submucosa, three muscularis layers, and L. serosa (not shown) as part of the peritoneum [47] (Fig. 4). During purification the connective tissue (submucosa) is saved while non collagenous parts are separated by mechanical and chemical means.

### Tendon

**Fig. 5:**
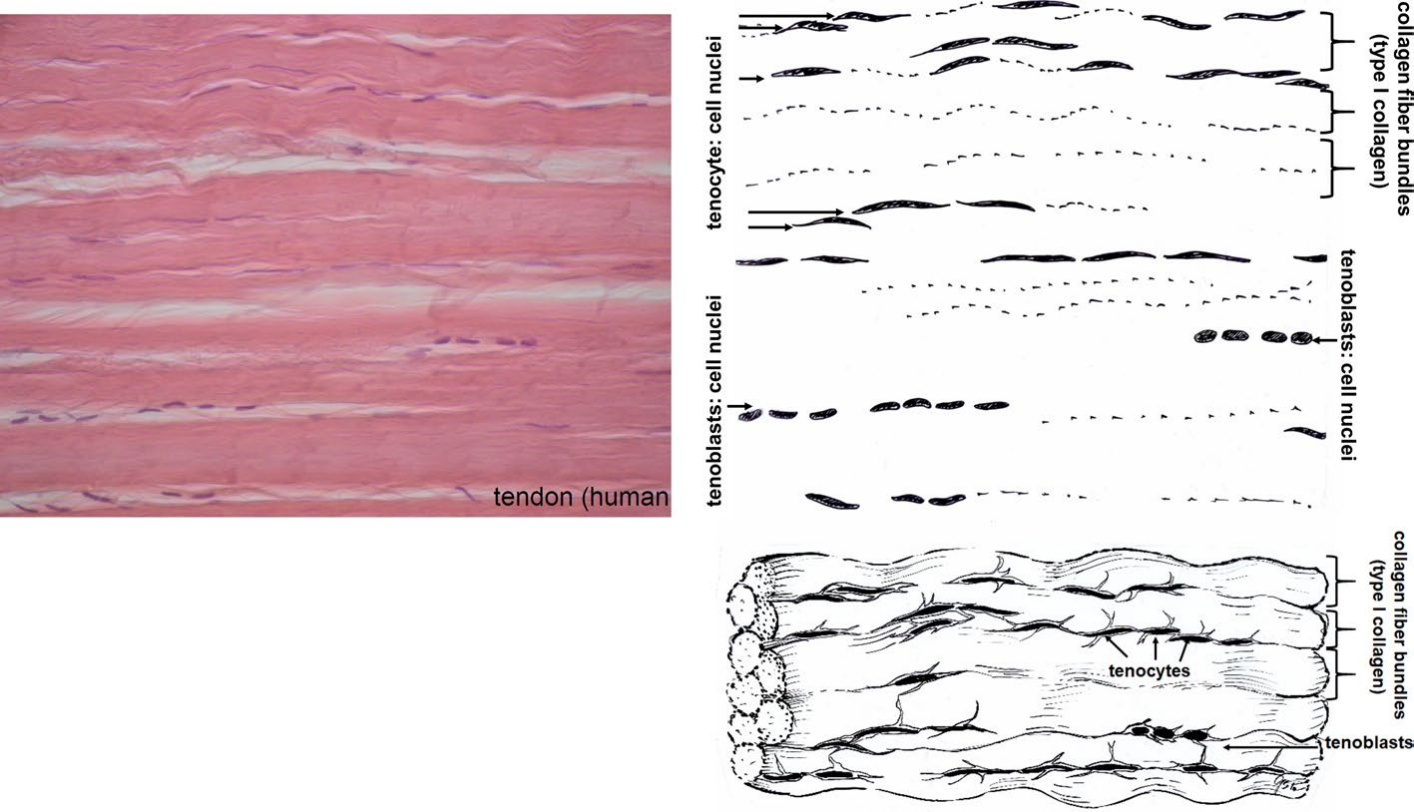
Histological cut (left) of human tendon and corresponding drawing. The fibre bundles are covered by an endotenon layer, the complete tendon by the epitenon (not visible). During processing the complete cellular components (tendoblasts and tendocytes) have to be removed

Tendons (Fig. 5) transfer mechanical load from muscle to bone. Therefore, tendons have to withstand high mechanical forces in the direction of pull. They are white coloured and show a fibrous texture with a low number of cells (tendocytes, tendoblasts) between the fibre bundles. Tendons are surrounded by an epitenon and diverse structures as retinaculae (cartilage covered floors), pulleys and sheaths which keep them in their anatomically correct position in rest and under load. Number and shape of these surrounding structures are different for different tendons e.g. Achilles tendon or the tendons of fingers and feet. Synovial sheath, bursae and paratenon are partly fibrous structures which reduce friction between tendon and their bony environment. The epitenon, a collagenous structure of thin transversal, oblique, and longitudinal fibrils of 8–10 nm each encloses one whole tendon. The endotenon covers fibre bundles, holds them together and enables mutual gliding of the bundles. It consists of reticular connective tissue with a typical crisscross pattern and glucosaminoglycans which are highly hydrated. Endotenon is interspersed with blood vessels, nerves and lymphatics to provide the underlying collagen tissue.

The collagen fibres in the fascicles of tendon are crimped and oriented longitudinally, but also transversally and horizontally, crossing each other. Therefore, load can be buffered in longitudinal direction, but to some extent also transversal, horizontal and rotational moves are transferred. Defining an exact size of the different levels of the structure of tendon (fibril, fibre, subfascicle, fascicle) is not reasonable because these sizes vary depending e.g. on anatomy, species and maybe individual variation [48–50].

### Recombinant sources

Currently collagen for biomaterials is extracted from human donators or xenogenic sources, mostly bovine, porcine or equine and it remains the gold standard to obtain collagen from these biological sources. Collagen and collagen peptides have also been produced from transgenic organisms, such as *E. coli*, yeast, mammalian cell culture, insects, and also plants [11, 51, 52]. The low yields, which correlate with high prices, limit the use and explain why such sources have not yet been established as alternative. Secondly, posttranslational modification such as hydroxylation of Pro and Lys in the right amount, glycosylation and the heterotrimeric compositions are challenges which have not completely been solved successfully [53, 54]. Therefore, it is obvious that animal sources remain the major source to manufacture collagen-based biomaterials for the next years.

## Collagen composition, formation, structure, stability and properties

The collagens comprise a family of animal derived fibrous glycoproteins. The composition of different collagen types and the resulting various structures have been a research topic since almost one century of researchers from all over the world. This will not be the key topic of the present review, because many others have summarized the basic knowledge about collagen and its versatility in building diverse complex structures that finally end up in the various structural different tissues [55–63]. However, a short summary will present some key features which appear to be important with regard to collagen processing by mechanical, thermal and chemical techniques.

### Collagen types

In vertebrates the collagens account for 28 different types coded by at least 45 different genes. Collagens are main structural components of connective tissue. Especially, in load bearing tissues such as tendon, bone, skin and cartilage the rope like proteins assume the load transmission. In these tissues only a small number of collagen types dominate. Collagen type I is the most abundant collagen in skin, tendon and bone beside the less frequent types III and V. Cartilage mainly consists of type II collagen. But, collagens are found in almost all tissues. Other types are arranged more like a network or like a rope of pearls, some types (FACIT) are associated in low amounts with the fibril forming types and others are found as part of membranes [55, 64, 65] (Table 2).

**Table 2:** Different collagen types are organized in various suprastructures

Supra structure	Collagen types
Fibril	I, II, III, V, XI, XXIV, XXVII
Fibril associated (FACIT)	IX, XII, XIV, XVI, XIX, XX, XXI, XXII
Network	IV, VI, VIII, X
Anchoring fibrils	VII
Transmembrane collagens	XIII, XVII, XXIII, XXV
Multiplexin	XV, XVIII

Type I dominates the tissues which are used as raw material for medical devices [55]

All collagens consist at least partly of triple helices which are formed by the same or very similar polypeptide chains leading to homo- or heterotrimers. Usually one collagen molecule is named monomer and the triple helix is called collagen molecule [66] in contrast to the nomenclature in polymer chemistry, where the monomer is the smallest unit before polymerization, e.g. ethylene/polyethylene or lactic acid/polylactic acid.

Key motif of all triple helical parts of the collagen molecules is the repeating glycine at every third position (Gly–X–Y)_n_ with X and Y being one of the 21 amino acids each. The amino acid composition of different collagen preparations of various tissues and species show only slight variation (Table 3). Specific actions of chemical treatments, which affect some amino acids such as Cys, Met (reduction/oxidation), Gln, Asn (alkaline treatment) or reactions with Lys and Hyl (chemical crosslinking), are discussed in the corresponding chapters.

**Table 3:** Comparison of the amino acid composition of different collagen preparations

Source	Bovine	Rat	Porcine	Equine	Human	Bovine	Bovine	Rat	Bovine	Bovine	Bovine
Tissue	Skin	Tail	Skin	Tendon	Meniscus	Pericard^b^	Skin^a^	Tail^a^	Skin^a^	In silico^a^	In silico^a^
Preparation	Soluble	Soluble	Soluble	Dispersion	Solid	Solid	Soluble^c^	Soluble	?	Type I	Type III
Asx	49	52	48	47	50	52	37	35	46	44	49
Hyp	92	91	81	90	88	99	95	91	89	104	139
Thr	19	23	19	19	18	17	16	21	19	18	13
Ser	34	40	31	33	32	30	32	39	34	35	44
Glx	82	84	75	79	76	68	67	68	78	72	69
Pro	122	115	130	131	112	113	131	121	131	115	95
Gly	309	302	321	331	316	321	340	332	318	330	348
Ala	109	105	112	92	115	102	107	105	103	114	86
Val	22	24	25	24	24	25	22	26	24	22	14
Met	7	7	7	8	9	5	6	7	7	6	9
Ile	14	13	12	12	15	15	12	12	12	11	14
Leu	29	30	29	27	30	34	23	27	26	25	15
Tyr	5	6	4	4	6	7	2	3	5	4	4
Phe	14	15	14	15	15	17	11	11	17	12	9
Hyl	8	8	6	10	11	8	13	12	6	6	5
Lys	30	30	30	23	24	28	27	27	24	28	31
His	5	6	5	6	7	8	5	5	5	4	8
Arg	51	50	51	52	50	51	55	55	52	51	47
Cys							1	1	2	0	2

^a^[505]

^b^ [506]; others: own measurements

^c^ pepsine soluble

Glycine (Gly) is the smallest amino acid and its hydrogen atom side chain always occupies the position in the centre of the triple helix, while X and Y are often proline and hydroxiproline and their side chains protrude from the central axis. In the Y positions of mammalian collagens almost all prolines (Pro) and some lysines (Lys) are hydroxylated. In skin two of these hydroxylysines per alpha chain are further enzymatically glycosylated prior to triple helix formation. This glycosylation is tissue specific and it is much higher e.g. in collagen of cornea or cartilage than in skin or tendon [67–69].

### Triple helix formation

**Fig. 6:**
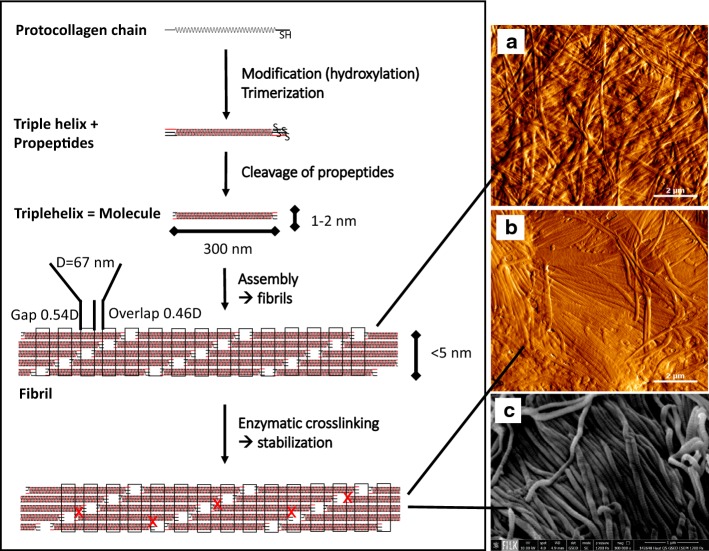
Schema: Monomeric protocollagen chains trimerize, the propeptides are cleaved off and the collagen molecules self-assemble to microfibrils and fibrils. Oxidation of lysine and hydroxylysine by lysyloxidase initiates the formation of the various natural enzyme-derived crosslinks [62, 508]. **a** Atomic force microscopic image (AFM) of reassembled collagen (dried); **b** AFM image and **c** scanning electron microscopic image of dried porcine skin splits (dried). AFM and REM images were prepared by Diana Voigt and Ralf Bittmann, FILK

The complete assembly of single protocollagen chains into triple helices is a key step of collagen formation and a complex intracellular procedure. Trimerisation begins at the C-terminal end of the chains, which are fixed by S-double bonds, and proceeds zipper-like in the same direction of each chain (Fig. 6 schema). It is initiated by additional globular non-collagenous domains which regulate mixture and orientation of the different collagen chains. After trimerisation the propeptides are cleaved off [49, 70–72].

Trimerisation requires all amino acids in trans configuration. Because the cis form of the iminoacids proline and hydroxiproline is energetically favoured, an isomerisation is required which is presumably enzymatically enhanced [65, 73]. In denatured non triple helical collagen 16% of X-Pro and 8% of X-Hyp were found to be in cis configuration. Because trimer formation is 1000 times faster than the cis–trans isomerisation, this isomerisation is the rate limiting step in collagen trimerisation [74]. In vitro denatured collagen reassembles only partly into triple helical structures. Nucleation begins randomly at different sites where the iminoacids are in cis configuration [75].

### Fibril assembly and formation of higher structures

Solved collagen molecules assemble into fibrils by an entropy driven process caused by the loss of solvent molecules which leads to an energetically minimized area/volume ratio [59]. In vivo fibrils show a spatially resolved organization and a defined stoichiometry of different collagen types (e.g. III and V combined with type I). Their assembly is thought to be cell and enzymatically supported by additional molecular organisers as fibronectin, integrins and minor collagens [60].

In vitro in buffering solutions, at neutral pH and temperatures > 20 °C, mammalian collagen assembles into microfibrils and fibrils leading to the typical cross-striation which is observed in electron microscopic and atomic force microscopic images (Fig. 6a–c). At deviating conditions (pH ≠ neutral, different salts, organic solvents) the collagen molecules solidify in a disordered structure as white or transparent precipitate.

### Natural crosslinks

Collagen is crosslinked intra- and inter-triplehelically. The natural crosslinks are formed by two different mechanisms. One is enzymatically controlled leading to specific divalent products which further react spontaneously to more complex, stable crosslinks. Enzymatically regulated crosslinking varies between different tissues [76]. The ε-aminogroup of telopeptidal lysine is oxidized by lysyloxidase to a carbanion which then reacts with lysine of the telopeptides to the aldimines dH-HLNL (dehydro-hydroxylysinonorleucine) and in the helical region to dH-LNL (dehydrolysinonorleucine). These Schiff bases are stable under physiological conditions but they are susceptible to acidic cleavage. Further, during maturation dH-HLNL reacts with histidine to histidino-hydroxylysinonorleucine (HHL) which is chemically stable. Telopeptidal hydroxylysine reacts to Schiff bases, which undergo an Amadori rearrangement, to form the stable ketoimines hydroxylysino-5-ketonorleucine (HLKNL) in the non-helical part of collagen molecules, and a lysine-5-ketonorleucine (LKNL), respectively in the helical part. Aldimines predominate in skin and tendon, in calcifying tissues and cartilage typically the ketoimines are found.

The second mechanism comprises a multitude of different spontaneous, not specific reactions, which are correlated to glucose and its oxidation products, leading to advanced glycation end products (AGEs) [77]. These AGEs became increasingly important with regard to aging mechanisms of tissues and organs, and diseases e.g. diabetes [76, 78–86]. Glucose, ribose and other sugars and sugar oxidation products react with lysine, hydroxylysine and arginine to form complex products from which only a few have been characterized to date [76, 87].

The maturated enzymatically induced crosslinks and the non enzymatic crosslinks cause the low solubility of collagen in buffers and weak acids of tissues from old aged animals and humans. They are very stable against enzymatic and chemical cleavage. This directly influences further processing as yield during dissolution, processing time and the kind of chemicals which are used.

### Elastin

Elastin is a fibrillar protein which is usually associated with collagen. It is found in different concentrations especially in blood vessels, ligaments and to some extent in skin [47, 88, 89]. In the mentioned tissues the content of elastin changes dependent on the topology. In ascending aortae the elastin content is higher than in descending aortae [47], in skin the content varies between grain and flesh side [90]. Concentrations up to 70% elastin are found in ligamentum nuchae of cattle and horses [91]. It assures the recovery of connective tissues under low load conditions and it endures billions of flexes without failing [13, 92, 93].

Elastin is composed of 72 kDa tropoelastin molecules. Similar to blockcopolymers these molecules consist of hydrophobic blocks, which coacervate directly after extrusion from the cell, and hydrophilic blocks, which are crosslinked by enzymatic control over desmosine and isodesmosine crosslinks including lysine [94]. The crosslinks highly stabilize the resulting network against hydrolysis. In contrast to triple helical collagen, which shows a distinct denaturation temperature, elastin has a glass transition temperature in fully hydrated state of 30 °C [95]. Elastin can be degraded by specific elastases while the collagen structure is saved [96]. In contrast, by treatment of elastin-rich tissue e.g. with hot 0.1 N sodium hydroxide for 50 min it was possible to extract pure elastin and to separate other proteins. This is only one possible method and others exist, e.g. treatment with BrCN in formic acid [97].

For a long time, elastin was tolerated as “contaminating” component during purification of tissues, which had not been specifically removed, but which did not mind the final use at all. On the one hand the exact elastin content was difficult to control, on the other hand elastin is very stable against degradation, and mechanical damage of the materials composites should be prevented. Recent developments add elastin as elastic component to prevent wound contraction (Matriderm^®^) to improve angiogenesis, and elastin is as well proposed to be used as sole biomaterial [13, 94, 98].

### Fibre architecture, orientation and mechanical stability

Tendon fibres, especially that of rats are easy to prepare and much more uniform than fibres of ligaments or even intestine and skin. Therefore, many ultrastructure studies in the past focussed on rat tail tendon collagen which was investigated such as tissue or reassembled soluble collagen. But some investigations of a broad range of tissues showed that fibril diameters and distribution vary depending on age, tissue, the layer in stratified tissue such as skin, and the species [99–101].

One of the main known functions of the collagen fibres is to take over mechanical load. The composition of the fibre distributions determines whether fibres may creep (smaller diameters e.g. lung, nerves or cornea) or transfer high loads (large diameters in tendons) [102]. The fibres are preferentially aligned in the main load bearing direction and they anticipate possible loads. Thus, the diameter of the fibres, the orientation of which differs tissue- and species-specific, plays an important role in tissue stability [40, 103–105].

In mammalian skin the fibres in dermis are oriented according to the Langer lines. Fibre bundles of collagen are connected by thin elastic fibres [106–111]. The collagen fibre network of the skin is denser at the back compared to the ventral side. Large differences of the thickness of fibre bundles are observed between papillaris and reticularis [33, 35, 112]. When skin is used as raw material for medical devices, this inhomogeneity causes different mechanical stabilities depending on sampling site and fibre orientation.

The collagen fibres in tendons are predominantly oriented longitudinally and to a much lower extent also transversely and horizontally. The longitudinal fibrils cross each other and form spirals and plaits. They are able to buffer longitudinal, and to some extent transversal, horizontal and rotational forces during movement [50]. Furthermore, tendon collagen fibres are crimped in contrast to skin fibres which are even. Small intestine submucosa (SIS) shows fibres in longitudinal orientation, the fibres of pericard and also urinary bladder are locally oriented but not over larger areas [45, 113, 114].

**Fig. 7:**
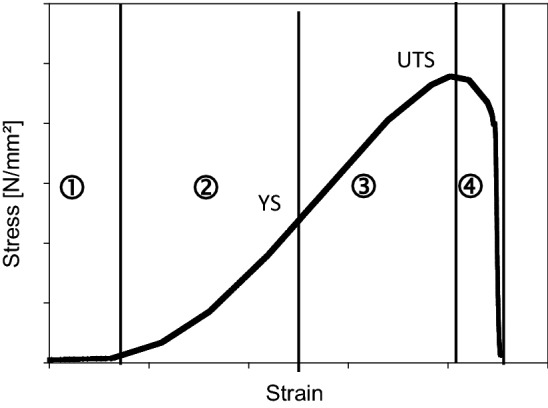
Stress-strain curve of the middle layer of wet porcine hide as an example for typical stress–strain behaviour of collagen tissues (own measured values). ① Toe region: Fibre and fibril crimps are straightened (tendon) and fibres are aligned (skin); the gap overlap ratio increase; resetting is caused by elastin fibres; ② Elastic region: Elastic fibres are deformed and fibres, fibrils and microfibrils begin to slide against each other; microfibrillar super twist causes torque transfer at crosslinks; molecules begin to stretch and to shear; ➂ Plastic region: Tissue begins to yield; interfacial delamination; plastic fibres deform and slide against each other; partial disruption of entanglements; exudation of bound water; helices uncoil and slide against each other. ④ Rupture: Tissue delaminates (skin) and disrupts in layer; fibres, fibrils and microfibrils defibrillate, disrupt and pull out; crosslinks between the molecules disrupt. YS, yield strain; UTS, ultimate tensile strength; [116, 509]

On which structural level which mechanical stability is generated is still intensively discussed. Stress–strain curves of collagenous tissues are typically S-shaped [Fig. 7; (1) to (4)]. Several authors [115–117] could show that each part of the curve reflects different structural effects at different structural levels.

In the toe to heel region of such curve (1) the whole structure is straightened at low strain. The network of collagen molecules is arranged in parallel e.g. in skin. In tendon and pericardium the zig–zag shaped parallel oriented molecules are lengthened. Kinks on every structural level such as fibres, fibrils, and molecules are drawn out. The elastin network stores the energy and resets after unloading [41, 116, 118, 119].

At increasing strain during elastic deformation (2) the slope becomes linear and the collagen takes over the applied forces. Collagen prevents the elastin network from overloading and stores the energy as well by elastic regions of the collagen fibres. The properties depend on the fibre composition and architecture which is regulated among other things by proteoglycans [120, 121].

Higher loads let the collagen molecules slip against each other (3), the fibres become stretched which then results in variations of the D-bands. Finally, fibres and crosslinks begin to crack (4). Depending on the tissue (e.g. skin) this cracking is often observed in layers, as not every layer shows the same elasticity. During tension measurement the tension curves then decrease not abruptly but stepwise with slight oscillation [42, 104, 115].

The physical stability of connective tissues relies on the network of fibrous proteins. In hydrated tissues the crosslinking density (reducible, mature crosslinks) determines whether a tissue behaves more brittle or is able to creep [102]. Synthetic crosslinks especially introduced by chemical agents are known to stabilize tissue additionally in wet state [122–125].

In recent years, computer modelling of the mechanical behaviour was increasingly used to simulate and predict the behaviour of collagen molecules and fibrils under load in wet and dry state. The data are based on collagen-like peptides, because for molecular dynamic simulations of complete collagen molecules and more complex higher structures the computing power is still not sufficient. It was found that the amino acid sequence substantially influences the mechanical properties of single tropocollagen molecules. These mechanical properties are not homogenously distributed along the collagen molecules, but show blocks of lower and blocks of higher stiffness which may result in concentrations of stress under load only in some regions. This has also been proposed to be the link between genetics, biochemistry and biomechanics [126, 127]. The principle of softer and stiffer regions is found again on the fibril level reflected by kinks [128] and again on the tissue level as zig-zag shape in tendon or non-parallel fibre orientation in skin (see above). Therefore, it can be assumed that this is an important principle presumably to regulate the elastic and plastic properties of the ECM.

Computer modelling but also other methods as AFM, light scattering and X-ray diffraction have also been used to calculate the mechanical properties such as stiffness and tensile strength of collagen-like peptides. For collagen molecules a stiffness of ~ 5 ± 2 GPa has been found, for microfibrils in wet state ~ 0.6 ± 0.2 GPa and for dry microfibrils 3.3 (2–7) GPa [126, 129]. Goh et al. [116] comprehensively reviewed the “Hierarchical mechanics of connective tissues” and summarized the physical properties for whole tissues in comparison to collagen fibrils and collagen molecules. The mechanical values e.g. stiffness and yield stress of tissues, fibre bundles (fascicles), fibrils, microfibrils and collagen molecules, which they collected from literature, showed very broad ranges (50–100% mean deviation) on every hierarchical level (Table 4). Surely, this high deviation is caused by different measuring conditions and also the broad range of tissues which were considered. However, the collection shows that the fibre bundles are the most flexible structural unit, slightly more flexible than whole tissue. But the fibre bundles are 4 times less stiff than microfibrils and 20 times less stiff than a collagen molecule. Similar factors between the different structural levels have also been observed for the yield strength.

**Table 4:** Stiffness and strength values of the different hierarchial levels summarized by Goh et al. [116], sponge by Jain 1988 [504], film by Koide and Daito [250], threads by Pins et al. [503]

	Stiffness (young module) (Mpa)							Yield strength (Mpa)			Molecule
	Tissue	Bundle	Fibril	Molecule	Film	Sponge	Thread	Tissue	Bundle	Fibril	
Average	701	276	1192	5150	1000	0.014	500	61	22	212	629
Mean absolute deviation	318	132	905	1653				18	10	148	589
Molecule: x	7.4	18.7	4.3	1.0	5.2	370000	10	10.4	28.7	3.0	1.0

Molecule: x is the factor of the physical properties of collagen molecules compared to the presented structure

### Thermal stability of collagen

**Fig. 8:**
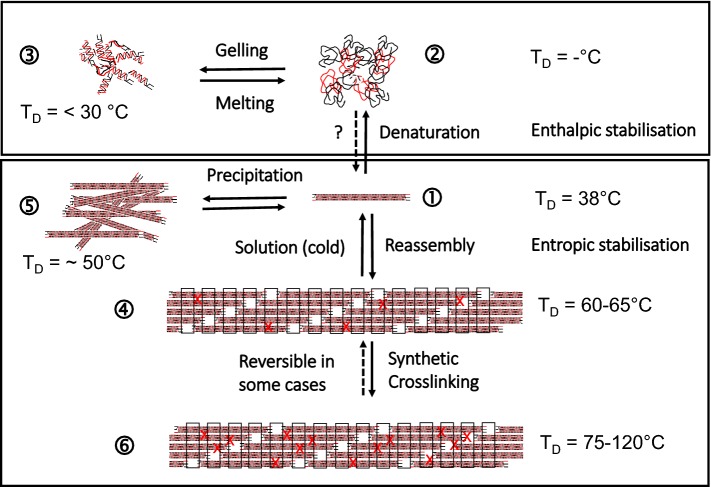
Structures of different collagen materials and derivatives and the corresponding denaturation temperatures T_D_. (1) soluble collagen; (2) gelatine solution; (3) gelatine gel; (4) reassembled fibrous collagen/tissue; (5) disordered precipitated collagen; (6) crosslinked (X) assembly/tissue; T_D_. Shrinkage temperature/denaturation temperature/melting (fully hydrated)

Processing of collagen requires to discuss the thermal stability of different structural levels of collagen. Heating of collagen leads to uncoupling of the triple helices at a typical temperature which is called denaturation temperature T_D_ and can be measured by differential scanning calorimetry (DSC) [130]. T_D_ differs between the different processing states or organization levels of collagen material [Fig. 8 (1) to (6)] and it directly correlates with the enzymatic degradation behaviour, the mechanical stability in wet state, antigenic properties, and the interaction with cells. T_D_ of fully hydrated soluble collagen (1) is near body temperature of mammals [131]. The absolute value deviates between mammals and poikilotherms [132]. Soluble collagen of warm-blooded animals shows denaturation temperatures of 36–40 °C. Poikilotherms can have lower denaturation temperatures adjusted to their habitats, e.g. coldwater fish such as antarctic ice fish shows a T_D_ of 6 °C. The hydroxiproline content correlates with T_D_ and it is assumed that the thermal stability of collagen is adjusted by the hydroxylation intensity of proline [67, 133].

The thermal stability of triple helical collagen molecules is markedly higher than that of globular proteins and the reasons for this have been disputed controversially for many years [62, 134–137]. Though many different ideas were formulated about the stabilising principles of the triple helix, such as hydrogen bonds, electrostatic interactions, van der Waals interactions but also hydrophobic interactions and stereoelectronic effects, it is now mostly accepted that a ladder of hydrogen bonds internally stabilizes the fully hydrated collagen triple helix to around one-fifth. X-ray diffraction studies in the middle of the 20th century and further biochemical and physicochemical investigations showed that these stabilizing bonds are located between N–H of glycine (Gly) and the C=O of other amino acids in X-position of the following strand [138].

The triple helices are surrounded by a network of water molecules that increases the thermal stability of the triple helices and supports their high denaturation enthalpy ΔH_D_ [137, 139, 140]. As T_D_ also ΔH_D_ can be measured by calorimetric measurements and it is assumed that ΔH_D_ directly reflects the number of hydrogen bonds, while T_D_ reflects an entropic and an enthalpic contribution to the stability of the collagen triple helix. The exact enthalpic and entropic part to T_D_ is not easy to estimate and to explain [133]. However, the denaturation enthalpy ΔH_D_ is a sensitive parameter of the degree of triple helical structure. For soluble samples this degree can also be estimated by polarimetry measurement that of insoluble samples is usually measured by calorimetry.

The hydroxyl group of hydroxyproline (Hyp) has an important influence on the stabilising principle of the triple helix, because a species-dependent increase of Hyp can be correlated to an increase in T_D_ and ΔH_D_ [130]. While most investigations only rendered indirect hints regarding such water network [133, 141, 142], Bella et al. [143] could measure, model and calculate the surrounding water structure for collagen-like peptides. It was deduced that water molecules additionally stabilize the triple helical structure though this was not possible to prove until today.

The entropy-driven assembly of collagen (4) increases T_D_ by approx. 20 K to achieve values of around 60 °C for skin and tendon collagen of mammals. Additional crosslinking (6) further increases the thermal stability: glucose-based crosslinking during maturation by several degrees [77, 87, 144] and synthetic chemical agents by 15 K up to 60 K and more. Assembly and disassembly of non-crosslinked collagen molecules is reversible [(1) ⇔ (4)] and likewise the differences of T_D_, while ΔH_D_ remains unaffected. Disordered precipitation of soluble collagen (5) also increases T_D_ but not as much as it can be achieved by an ordered reassembly (4).

By application of temperatures higher than T_D_ or by addition of hydrotropic agents at ambient temperature (e.g. Urea, LiBr, SCN^−^) collagen molecules in solution denature into single protein strands. In contrast to the assembly and reassembly of collagen molecules into fibrils the denaturation of the triple helix of soluble collagen is only partly reversible leading to physical gels [(1) ⇎ (2); (2) ⇔ (3)]. The complete formation of collagen molecules from single chains in vitro seems to be only possible under restricted conditions [145–149].

Gelatine is a hydrolysate of collagen, which is manufactured by topochemical hydrolysis, that means selective cleavage of specific bonds to make the collagenous tissue soluble [150]. In contrast to denatured soluble collagen gelatines show broad molecular weight distributions. The gelation of gelatine solutions by cooling [(2) ⇔ (3)] is interpreted as imperfect reassembly of the collagen chains. Firstly, this only incomplete trimerization in vitro is presumably caused by these broad molecular weight distributions of the gelatine molecules and a “contamination” with process-induced collagen peptides and aggregates which markedly deviate from the distinct peaks of original collagen molecules [151, 152]. Secondly, the chemical structure of collagen especially during the gelatine process is modified through desamidation of Gln and Asn [153]. Not least, the gelling rate of gelatine gels depends on the isomerization of the iminoacids in cis configuration to trans [154] and the triple helix content of gelatine gels is increasing over months [152]. Therefore, the melting temperature of a gelatine gel is always lower than T_D_ of the corresponding collagen molecules (3).

**Fig. 9:**
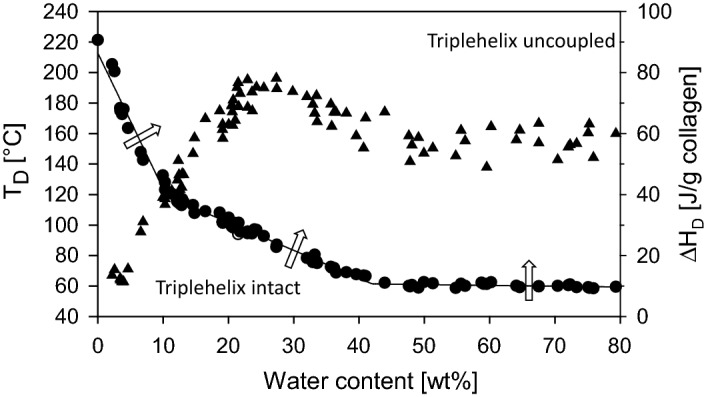
Denaturation temperature T_D_ (filled circle) and denaturation enthalpy ΔH_D_ (filled triangle) of decellularized bovine skin at different water contents. If T_D_ is exceeded (arrows) collagen triple helices uncouple. At dry conditions T_D_ increases, while ΔH_D_ decreases [156]

Upon drying T_D_ increases and ΔH_D_ decreases. This shows that the entropic contribution to T_D_ becomes more and more important, while the number of hydrogen bonds is decreased by water removal lower than ~ 15 wt% (Fig. 9). Almost water-free collagen shows T_D_ of up to 220 °C, while the denaturation enthalpies decrease up to a minimum value of one-fifth of the value of fully hydrated collagen. This is consistent with the enthalpic part of the hydrogen bonds in the center of the triple helix. The increasing entropic part correlates with the decreasing mobility of the collagen molecules. It predominates more and more upon increasing dehydration of collagen [142, 155, 156]. The denaturation temperatures and the enthalpies give important hints about the organization degree and the nativity of collagen. It allows to control processes and to prevent denaturation.

### Diffusion and adsorption

Diffusion and adsorption of chemical additives and their reaction kinetics have to be considered intensively, when collagenous tissues such as skin, tendon, pericardium or small intestine are purified from non-collagenous material: (1) the tight fibrous structure as well as alternating layers of different tissue structures are massive obstacles for molecular diffusion. (2) The tissue structure separates molecules regarding their hydrodynamic volume which corresponds to their molecular weight. (3) As any protein and in contrast to many other natural polymers, collagen acts as an exchanger for anions, cations including protons, hydrophilic, and hydrophobic substances, and (4) the exchange of collagen depends on pH and temperature.

Diffusion, especially through skin and intestine, is not only important for processing, but it is as well an important parameter for the cosmetics and the pharmaceutical industry. To describe the permeability of substances through e.g. skin or intestine in vitro and in vivo, many different models were developed. These are partly mathematical models with references to chemical properties (hydrophobicity, molecular weight), or mechanistic models (brick and mortar models) which assume different mechanisms of permeation through hydrophobic cell layers. Currently, the published models neglect mixture of substances e.g. hydrophobic additives emulsified with surfactants, or combinations of differently hydrophobic substances and their partial coefficients during application [157–165]. For skin, these models assume the Stratum Corneum consisting mainly of dead keratinocytes and keratin as strongest barrier. The intestine models consider the mucosa and active membrane transports.

When tissues are processed to manufacture biomaterials, the non collagenous parts are removed as completely as possible. Often collagen raw materials are processed in an excess of aqueous floats in moving drums or stirred reaction vessels with built-in baffles. To avoid mechanical stress, collagenous tissues are also fixed in batch vessels, or purification fluids are only pumped past in a circle. Processing lasts from a few hours to several days at temperatures between 5 and 30 °C. In these agitated systems, the float can be assumed as ideally mixed already a few minutes after addition of chemicals. Therefore, a first approach does not have to discuss diffusion in the float in contrast to diffusion through the tissue structures, because of the forced agitation [20, 166].

**Fig. 10:**
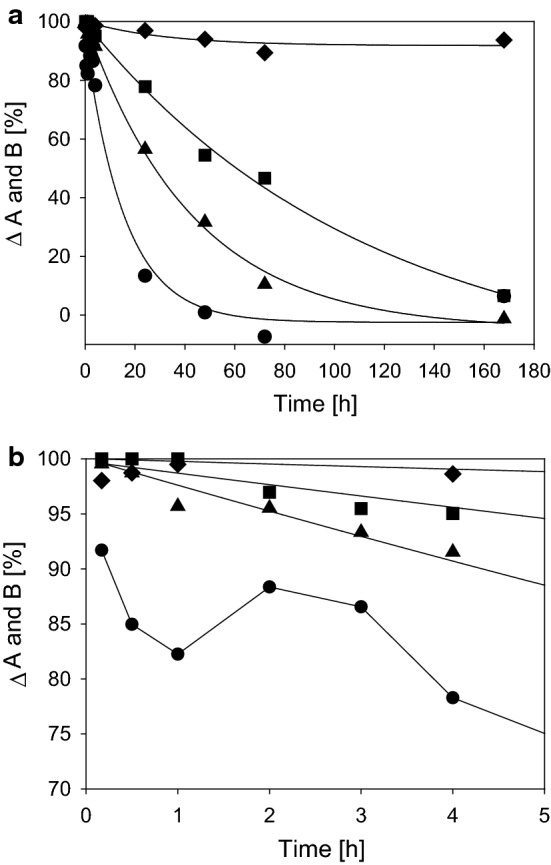
**a**, **b** Permeability of different substances through collagen membranes (Biogide^®^) prepared from porcine skin. The diffusion rate between chambers A and B depends on molecular weight and the chemical nature of the substances (**a**). Sodium chloride is absorbed at low concentrations until saturation (**b**). Filled triangle: Tryptophane; filled circle: NaCl; filled square: glucose; filled diamond: hyaluronic acid (M_w_ ~ 1.2 MDa); (own unpublished data)

The collagen structure shows a cutoff for different molecular weights. Own unpublished results in a diffusion chamber system (two agitated chambers which were separated by a collagen membrane as described in [167]) demonstrated (Fig. 10a) that sodium chloride diffuses very fast through a commercial porcine dermis based membrane (Biogide^®^), while hyaluronic acid (molecular weight M_w_ ~ 1.2 MDa) is widely excluded. A similar model was also used to investigate the cutoff of more complex diffusion barriers, such as collagen sponges and collagen threads, which confirmed this observation [168]. Ho et al. [169] used films of soluble collagen which had been crosslinked and found that the crosslinking degree affects the diffusion behaviour.

For a low quantity of permeating molecules collagen behaves like an exchanger. Glucose (M_w_ 180 g/mol) with many hydroxyl groups diffused slower through the investigated Biogide^®^ membrane than tryptophan (M_w_ 204 g/mol) which is a hydrophobic amino acid but of similar molecular weight as glucose. Adsorption was observed for ions such as sodium chloride (Fig. 10b), and other authors observed adsorption for calcium ions [166, 170], surfactants [167, 171, 172], growth factors [173–176], but also endotoxins [177–180]. Low concentrations of adsorbed molecules can only be removed with high amounts of washing floats and by an excess quantity of molecules which are able to replace (ions, protons) or bind (chelating agents) the unwanted adsorbate.

The structure of the raw materials is usually not homogenous. Skin as an example is highly asymmetric and thick enough that diffusion becomes a relevant parameter. The asymmetry leads to different diffusion coefficients from both sides that has to be considered when processing agents diffuse into and out of the skin from both sides.

When treating thin collagen materials such as intestine submucosa or pericardium, diffusion plays only a minor role. Big tendons (e.g. tendo calcaneus from equine, bovine or porcine sources) in contrast have to be treated for long periods of time to achieve sufficient diffusion. Not least, organ decellularization is a particular challenge, because a laminar flow in the vessels has to be assumed. Diffusion then occurs similar to the in vivo situation across the membranes of vessels, and large washing volumes are necessary to remove all non-structural material. Because of the complex architecture of the vessel trees and their unpredictable hydrodynamic behaviour, it is very difficult to predict or model diffusion scenarios. However, to optimise the cleaning procedures, this would be very important.

### Reaction kinetics

Reaction kinetics were investigated and calculated for the structural transformations of collagen such as for denaturation and fibre assembly as well as for many different chemical reactions e.g. crosslinking and modifications of collagen and of gelatine (denatured collagen). The different kinetics not only depend on the temperature, but also on pH and additives like salts or organic components, and the kind and amount of solvent. Not least, there is an interaction between the different kinetics. Table 5 compares the reaction rates of different reactions and structural transitions.

**Table 5:** Different kinetics have to be considered which influence the processing times during tissue decellularization, purification and processing

Time scale	T; T opt (°C)	Reaction rate			Remark	Refs.
		pH 3	pH 7	pH 12		
Denaturation	> T_D_	Seconds	Seconds to hours	Seconds	Depending on the degree of structural organization, crosslinking degree, solvent concentration and additives	[181, 480–483]
Renaturation	0 to ~ 20; 4 to 10	Hours to months	Hours to months	Hours to months	Dependent on the sequence, molecular length and covalent crosslinks	[152, 481, 484, 485]
Reassembly/fibrillation	10 to 38; 20 to 35	–	Minutes to hours	–	Only possible in neutral conditions	[186, 190, 191, 486–488]
Disassembly	< 37; < 5	Seconds	Minutes to hours	Seconds	At pH deviating from the IEP a high swelling is observed which can be assumed as disassembly [343]	[189, 191, 487]
Crosslinking reaction		Seconds to hours	Seconds to hours	Seconds to hours	Highly dependent on the kind of reaction	[192, 248, 420, 421, 489–491]
Diffusion		Hours	Minutes to hours	Hours	See special section	

The denaturation process of the collagen triple helix (helix ↔ coil) in solution had been discussed controversially over decades. Miles [181] assumed an irreversible rate process, while others interpreted the calorimetric results as an equilibrium of monomers and trimers [58, 134, 136, 148]. Denaturation is a fast process which lasts only in the range of seconds if the collagen molecules are separated (pH ≠ IEP).

Complete renaturation of the triple helices is possible in idealised systems (collagen type III, crosslinked in one position by disulfide bonds, diluted solution, long reaction rate). It is hindered by cis–trans transitions, structural features and partial cleavages [134, 148]. The renaturation kinetics is determined by the cis–trans transitions and is a slow process.

Gelatine is a denatured, partially cleaved collagen with very broad molecular weight distributions which often still contains crosslinked particular components resulting from the manufacturing process. Therefore, the setting rate, which corresponds to the renaturation of collagen triple helices, is much slower than that of idealised collagen. Setting begins very fast by cooling a gelatine solution, but does not end in practically measurable time [152, 182–184].

In vitro collagen assembles into microfibrils and fibrils of varying length and thickness which depends on temperature, pH, and additives [185, 186]. Fibrillation only occurs in the isoelectric range with the highest fibrillation rate in the range of minutes at pH 9.2 in saline phosphate buffer (PBS) [187]. Not only the rate of assembly but also distributions of fibre length and thickness depend on further additives (e.g. glycine) and temperature [188].

Disassembly of reassembled collagen occurs when the fibres are exposed to lower temperatures. The higher the cooling rate the faster is the disassembly. However, some small filaments remain. In contrast to earlier investigations, which assumed crosslinking as a reason of these stable aggregates [189, 190], de Wild et al. [191] assumed that they are stabilized kinetically, because the stable aggregates can be completely dissolved again in acid.

Finally, collagen and gelatine are widely modified and stabilized by versatile chemical reactions which comprise almost all possible reactions of the side chains, especially amine (Lys) and carboxylic groups (Glu, Asp). Examples of possible reactants are mentioned below (section crosslinking). Reaction times and conditions are as manifold as the reactions and a comprehensive consideration is beyond the focus of the present review. From the practical point of view, the reaction times have to be adapted to the manufacturing processes which usually do not last longer than hours. But the reaction time must not be too fast, for diffusion and equilibration to be still possible. As examples, aldols or condensed tannins often show reaction rates of several hours up to days, while aldehydes may react in less than seconds [192–196].

In collagen tissues, which comprise complex structures, the chemical reaction always competes with diffusion of chemicals in the tissues. Therefore, there is no easy rule about which process is favoured—diffusion or chemical reaction. Uncoupling of these sub-processes can be achieved most easily by adjusting the pH. Possible reactive groups (e.g. –NH_2_) are only available as non reactive (–NH_3_^+^) at acidic pH until diffusion of the chemicals achieved an equilibrium. The same applies to alkaline pH and carboxylic side chains of the amino acids. By cautious adjustment of the pH the reaction can be regulated.

### Molecular weight distributions and particle sizes

The molecular weights of human type I collagen chains without propeptides (UniProt, P02452, P08123; Expasy ProtParam) are calculated to be 94.8 kDa (α1(I)) and 93.6 kDa (α2(I)). The calculated molecular weight of a collagen molecule would then be 283 kDa. When the molecular weight of the collagen chains type I was measured by SEC-MALS, it was found to be 92 kDa that of one denatured triple helix around 300 kDa. A chromatographic separation of α1(I) and α2(I) chains was not possible [151]. Other methods of protein chemistry as SDS-PAGE allow to separate the different collagen chains and also specific peptides [197–199]. In tissue the collagen is crosslinked intra- and intertriplehelically by covalent bonds. The number of crosslinks increases with age [77]. This results in extremely high molecular weights which cannot be measured anymore. Table 6 shows the molecular weights of differently processed materials. Permacol^®^ and Xenoderm^®^ are decellularized tissues whose connective tissue architecture is saved. In contrast, Surgicoll^®^ and Resodont^®^ are manufactured from purified wet ground tissue.

**Table 6:** Different commercial forms of collagen materials and their properties solubility under different conditions, their estimated molecular weight and its distribution, helical degree and estimated crosslinking content

Material	Example	Possible supplier	Solubility (aqueous saline buffer 0.1% < 40 °C)	Solubility (aqueous saline buffer 0.1% > 40 °C)	Solubility (acetic acid 0.1 M < 15 °C)	Average molecular weight	Molecular weight distribution	Crosslinking content	Triple helix content native (+) denatured (−)
HMDI crosslinked porcine skin	Permacol	Covidien	−	−	−	∞	∞	++++	+
Lyophilised porcine skin	Xenoderm	MBP	−	−	−	∞	∞	++	+
Lyophilised sponge from porcine skin dispersion	Surgicoll	MBP	−	−	−	∞	∞	++	+
Convection dried sheet from equine tendon dispersion	Resodont	Resorba	−	−	−	∞	∞	++	+
Denatured powder from skin collagen	Thermoplastic collagen	FILK	−	±	−	Several millions	++++	+	−
Acidic extract (cold) from calf skin	Soluble collagen	Symatese	+	+	+	270 kDa	+	±	+
Aqueous extract (hot) from limed skin or bone	Gelatine	Gelita	±	+	±	Ca. 50–150 kDa	+++	−	−
Enzymatically treated extract (hot) from skin or bone	Collagen hydrolysate	Gelita	+	+	+	Ca. 5–20 kDa	++	−	−

Wet grinding is an established technology to prepare collagen suspensions for the food industry and to prepare e.g. collagen sponges and membranes for medical applications. It is very difficult to determine particle sizes of wet ground collagen, because of the shape of the particles, intensive interactions of the particles themselves, and the low contrast between the particles and the usually water-based dispersing medium. The microscopic appearance in swollen state has been found to be fibrous, lobe-shaped, partly solved partly gel-like, and hence neither homogenous in size nor in shape. Some correlations to the grinding degree are found by ultracentrifugation, by microscopy or by particle counting. In contrast to chromatographic procedures or particle size measurements a repetitious accuracy is difficult to achieve, not least because physical entanglements and physicochemical interactions cannot be excluded completely. The moment of measurement only reflects a single observation which is again markedly varied by pH, ions and water content. Therefore, in industrial processes, especially the viscosity is measured to evaluate the processability [200–202].

If collagen tissue is ground in dry state, the smallest particle size is in the range of a hundred microns or larger. The particles appear as fragments of fibres [203, 204]. Partly denatured tissue can be ground into much smaller sphere-like particles which can be processed by thermoplastic machines (thermoplastic collagen) [205].

Very broad molecular weight distributions of molecular disperse collagen preparations accrue by combined thermal and chemical degradation of tissue e.g. during gelatine manufacture. They range from particular components with M_w_ > 10^7^ up to < 10^3^ Da. Depending on how the tissue is processed, distinct peaks (gelatine type B) or broad peakless distributions (gelatine type A) are observed [151, 152, 206, 207].

## Processing collagen—the toolbox

**Fig. 11:**
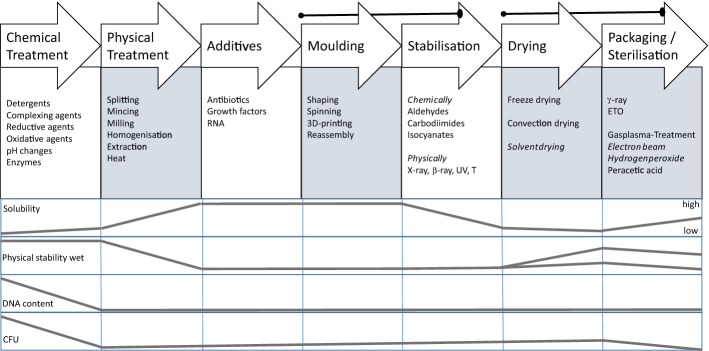
State of the art of collagen processing technologies, their processing steps and their effect on the parameters solubility, physical stability, DNA content and colony forming units (CFU). The brackets connect process steps which are usually combined in this order

Processing of collagen materials is organized as a sequence of technological steps lasting several hours up to several days each. Many of these steps require manual handling and often the degree of automatization is low. The technological steps comprise mechanical, chemical and physical treatments [3, 4, 20, 21, 208, 209]. Figure 11 presents the principal combination of such technological steps as a sequence, applied chemical agents or variants of physical treatments and their effects on the important parameters solubility, physical stability in wet state, the DNA content and the microbiological quality (CFU—colony forming units). To manufacture collagen-based biomaterials, tissue has to be purified, (disintegrated, reshaped, stabilized, dried), packed and sterilized. The steps in brackets are optional. The effects of the several chemical agents on the collagen structure are discussed in the following chapter in more detail.

The order of the steps is not necessarily fixed but it is often used as shown. *Stabilization* (crosslinking) is not only used to adjust degradation in vivo but usually follows *shaping* to fix the new form. *Packaging* is enqueued as last step to market a sterile and save product [210]. Sometimes sterilization is performed at earlier stages, especially when wet products are produced which then cause aseptical downstream processing and packaging [210].

By application of different variants of the mentioned series of technological steps, such different materials are prepared as for instance decellularized membranes from dermis, pericardium, small intestine or urinary bladder, injectable solution, injectable suspension, membranes from tendon and minced skin, powder, and sponges and finally hydrolysed, denatured collagen [1, 3, 7, 27, 208, 209, 211].

**Fig. 12:**
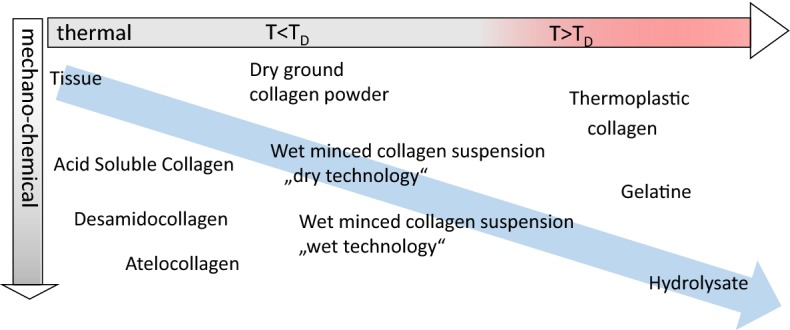
Collagen materials manufactured by different technologies. The blue arrow marks the degree of desintegration of the collagen structure, the red range a thermal treatment of the material higher than the T_D_

Figure 12 discriminates the field of collagen-derived materials in two main processing directions. One direction comprises mechano–chemical processes, the second one shows thermal denaturation. Both directions lead to fundamentally different materials. Physico–chemical extraction from tissue saves the triple helical structure (acid soluble collagen) and chemical (Desamidocollagen) and enzymatic treatment (Atelocollagen) comprise modified triple helical collagen molecules.

Solubility of tissue increases slightly by chemical treatment but markedly by physical disintegration. Reciprocally the physical stability decreases by mechanical disintegration. Wadding-like collagen powder (dry grinding) or suspensions (wet mincing) are achieved by mechanical disintegration. It is necessary to mention that prior to these mechanical disintegration steps the tissues have to be purified as much as possible. Long-lasting chemical treatment and especially thermal treatment, which affects the structure of collagen, is prevented, however. The powders reflect collagen fibres and fibre bundles which collapsed by drying. Impurities are completely encapsulated and removing them becomes impossible. Collagen minced in wet state is highly viscous. Furthermore, diffusion is very slow and even pH adjustment in small volumes (millilitre to litre) usually lasts several hours up to days.

Thermal treatment uncouples the triple helices. It is again necessary to purify the raw material as much as possible prior to thermal treatment. While thermoplastic collagen describes an intermediate which sustained only a short treatment with chemicals and thermal uncoupling of the triple helices [205], gelatine and hydrolysates are intensively processed by chemical, mechanical and heat treatment. The latter both are soluble products which can be easily purified by many different techniques such as filtration and chromatographic methods as well [212, 213].

In addition to the molecular weights, Table 6 shows other properties of the differently processed materials. Every material except thermoplastic collagen also reflects marketed products. It is obvious that the solubility increases with the degradation degree and that the molecular weight decreases, accordingly. Crosslinking prevents solubility and crosslinked samples often show very high molecular weights. The triple helix content directly correlates with a heat treatment that exceeds T_D_.

**Fig. 13:**
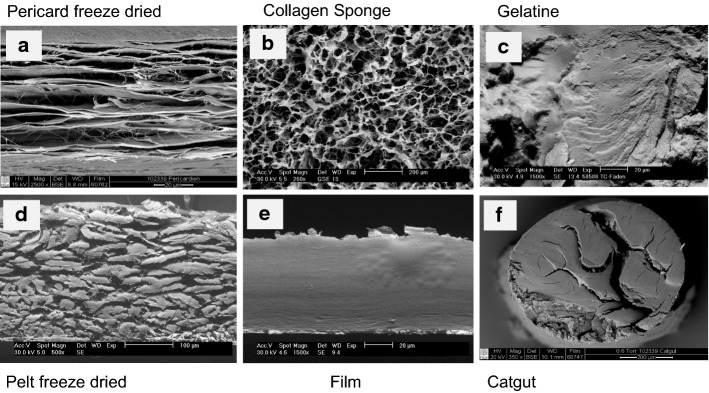
Comparison of microscopic structures of principally different processed materials. Pericardium (**a**) and skin (**d**) are decellularized and freeze-dried, for sponge (**b**) purified skin is minced and freeze-dried, for films convection-dried (**e**), instead. Catgut (**f**) consists of purified small intestine (SIS) which is cut in strands, twisted, stabilized by crosslinking, and convection-dried. Gelatine (**c**) is hydrolyzed collagen of skin or bone which have been convection-dried

Figure 13a–f compare the scanning electron microscopic appearance of a selection of the different materials manufactured by variants of the processing sequence [209]. Freeze-dried decellularized pericardium (Fig. 13a) and unhaired decellularized skin (Fig. 13d) reflect the corresponding structures of the raw tissues. While the pericardial fibrosa delaminates to some extent, in skin the collagen fibre bundles agglutinate. Catgut (Fig. 13f) is manufactured from purified SIS which is cut in small strands, twisted and convection dried under tension. A common technique to manufacture intermediates is to decellularize hide followed by homogenization in wet state, and drying. Porous sponges (Fig. 13b) are achieved by freeze drying, and compact films (Fig. 13d) by convection drying. Not least, gelatine is the only material in this series of microscopic pictures which reflects denatured collagen (Fig. 13c). Microscopically no difference can be observed between the film in which the triple helices are still present, and gelatine, however.

The step *chemical treatment* (Fig. 11) is commonly the rate-limiting step of the whole process. Processing times depend on concentration and concentration gradients of agents, treatment temperatures, and the intensity of washing, which directly correlate with diffusion and reaction times. The processing times vary considerably between different tissues, their structure and the treated volumes. While skin and tendon are thick and compact, pericardium is much thinner but compact as well, and small intestine is thin and rather elastic. Table 7 compares selected published processing times for different materials. Many more processes have been applied for patents [210] and it is not easy to find realistic values. The published times vary in a broad range even for the same tissue and it seems that there is serious potential for optimisation which is not directly correlated to the specifications and limits.

**Table 7:** Frame of wet technologies to decellularize diverse tissues

Tissue	Species	Form [2D; 3D]	Freeze/thaw cycles	Washing cycles	Surfactant	Acid/base	Enzyme treatment	Complexing agents	Mechanical treatment	Sterilisation /decontamin.	Time (h)	Refs.
				Water, buffer, salts	SDS, Triton X, others	Organic, inorganic	DNAse, protease	CHAPS, EDTA, etc				
SIS	Porcine	2D	+	125	13	−	−	−	(−)	−	138	[492]
Pericardium	Bovine	2D	−	1	−	3	−	−	−	Gamma	4	[46]
Tendon	Diverse	3D									5–48 (96)	[493]
Dermis	Human	2D	+	19	−	−	3	47	−	Gamma	69	[494]
Dermis	Porcine	2D	+	18***	22**	−	6	(+)	(+)	PAA; H_2_O_2_*	49	[495]
Dermis	Porcine	Dispers	+	4	2	8	−	−	+	−	13	[496]
Dermis	Porcine	Dispers	+	35	−	39	−	−	+	H_2_O_2_	74	Own trials, unpublished

The numbers reflect hours of treatment

as, antiseptic processing; ab, addition of antibiotics

* Additionally other sterilization techniques were investigated

** Complexing agents and surfactant are applied together

*** Washing comprised 12 h ethanol treatment

### Examples—selected technologies varying physical, thermal and enzymatic stability

Processing connective tissue causes structure downgrading to different extents by mechanical, physical and chemical means. The resulting materials comprise stable tissue up to soluble collagen hydrolysates based on the same raw material collagen only differing in degree of destruction and depending on the mechanism of degradation (Fig. 12). To cover the entire range of possible collagen materials, five examples of different technologies and the resulting materials will be subsequently discussed, which vary fundamentally regarding their stability to temperature and enzymatic degradation.

#### Saved tissue structure—hernia implant

Decellularization usually aims to save the mechanical structure of the grown tissue. The mechanical properties shall be affected as little as possible. Mostly it is also aimed to remove all non-collagenous materials except elastin. This concerns e.g. globular proteins, bound glucose and galactose molecules, lipids, DNA, components of cell membrane, nuclei and if necessary processing agents. To some extent growth factors may be saved or removed as well. Nevertheless, after one or repeated freeze–thaw cycles, decellularization comprises an intensive treatment with complexing agents, surfactants, also alkaline and acids and possibly enzymes [3, 4, 20, 21]. By saving the structure of connective tissue skins, pericardium, small intestine and fascia are manufactured from human, porcine, bovine and equine sources (Epiflex^®^, Xenoderm^®^, Xenoguard^®^, Oasis^®^). Examples for marketed materials are listed in Table 1. To further stabilize these decellularized tissues to enzymatic degradation, they can be crosslinked by chemical means (Permacol^®^, CuffPatch™, Dura-Guard^®^).

The decellularization strategies fed the hope that complex structures of whole organs can be sufficiently purified and, if freed from any contaminants, recellularized for organ replacement. However, currently the quality of decellularization and its evaluation has to be approved and recellularization is still an important challenge [214–218].

#### Saved fibre bundles, fibres and triple helices—hemostyptic sponges and dental films

The technologies to decellularize tissue are used as well to purify raw materials which are further desintegrated to fibrous preparations. The subsequent key steps are grinding and mincing in the cold after intensive acidification of the tissue. The mechanical desintegration steps lead to suspensions with highly swollen gel-like collagen fibres. The triple helices remain largely intact, however. If skin or tendon is used as raw material the masses of which consist of more than 95% collagen, low amounts of fat and some non-collagenous proteins (especially elastin), they are still part of the system. When skin is used as raw material, such technologies often comprise very intensive treatments with strong acids and alkaline covering a pH range of pH 1 to pH 13 [219]. Then glucosaminoglycans and nucleic acids are removed completely during purification.

It is almost impossible to remove contaminations from these highly swollen gels. Extrudable masses show very high viscosity at dry matter contents of 5 to 10% and low diffusion rates of agents and even protons. Adjusting the pH lasts several hours, mixing and homogenization require high mechanical energy. A real homogenous distribution of chemical substances needs several days. Castable suspensions are achieved only at dilutions to 1% and lower. It is impossible to filter these suspensions on a molecular level and therefore it is convenient to remove as much as possible accompanying substances prior to the mechanical homogenization. By varying the viscosity, suspensions can be shaped easily into tubes, threads, films or sponge-like shaped articles of many dimensions [4, 200, 220–222], such as a loophole for the limited dimensions of decellularized tissues which correlate to the dimension of the source material. Marketed materials based on restructuring of fibrous suspensions are MBcollagen^®^, Matristypt^®^ or Parasorb^®^ to name examples.

#### Injectables and solid articles from reassembled soluble collagen

Soluble collagen is extracted by organic acid as described and filtrated to achieve sterility. These solutions reassemble to form gels by buffering acid soluble collagen in cold state and increasing the temperature to body temperature [223]. This principle has been used for a long time not only in vitro but also as injectable for soft tissue augmentation in situ (Zyderm^®^). Cold buffered soluble collagen was injected and reassembled in situ initiated by body temperature [3]. Reassembled collagen shows the typical cross-striation and fibre dimensions depending on the temperature, additives, buffering concentration, and the collagen source and preparation.

Soluble collagen can also be used to coat surfaces of cell culture well plates and to manufacture films, and it was used to prepare more complex materials such as multiple layered sheets, tube-like structures and capsules [224]. Hard tissue replacement materials were manufactured by combined reassembly and silicification of pre-polymerized siloxanes or by combination of reassembled gels with hydroxyapatite [225–228]. Not least, soluble collagen can be spun by diverse techniques (see below). Disadvantage of soluble collagen is its comparably high price per gram when solid articles are manufactured.

#### Thermally treated, insoluble collagen

Fibrous collagen cannot be transferred into powder with spherical particles but only into wadding-like material. But, if the collagen is partly denatured, the fibrous structure collapses and allows to process the collagen in thermoplastic machines similar to synthetic polymers. To prepare this thermoplastic collagen (TC), skin is unhaired and decellularized. The collagen triple helix has to be denatured, which can be achieved by treatment in excess hot water (80 °C), by extrusion of the wet material (115°) in a microwave or by treatment in a drying oven in hot vapour. The material is then dried and ground into powder. This powder is prepared for extrusion by addition of water, glycerol and other additives [205, 229].

Because of its partial denaturation TC shows some properties of a gelatine. Dried material becomes gel-like when soaked in water. It takes up several hundred percent of water by swelling, and it is easily degradable by proteases. In contrast to gelatine, it is almost not soluble in warm water [205, 230–232].

In the presence of 15 to 20% of water, the material can be processed by extrusion into films, threads, tubes or by injection moulding into 3D articles, and this seems to be unique compared to other proteins [229]. By thermoplastic processing it can be mixed in broad ranges with synthetic polymers not only acting as filler but also as a second polymeric component. Furthermore, TC was mixed with hydroxyapatite to generate bone-like materials by thermoplastic processes. The resulting dry and machinable parts showed mechanical properties like bone. By exposure to saline buffering solution, the collagen became gel-like again. Materials made of thermoplastic collagen have not been tested in animal and clinical trials yet.

#### Medical applications of gelatine

Gelatine as well has some tradition in medical and associated applications. It is used as pharmaceutical auxiliary to produce capsules and microspheres, to coat surfaces of cell culture plates, to coat textile-based vascular implants, to prepare hemostyptic sponges, films for ophtamologic applications and finally plasma substitutes and adhesives [6, 233–237]. In contrast to the food industry, where the gelling ability of gelatine and its melting temperature of 20 °C to 25 °C are the most important properties [150], in medical applications the high biocompatibility is the main advantage. Gelatine can also be modified, e.g. succinylated to adjust the chemical resistance to aldehydic reactions, e.g. reducible sugars and plant-derived pharmaceuticals. Gelatine is crosslinked by many chemical agents as well as transglutaminase, and the enzymatic degradability is modified by additional stabilisation. In the field of materials prepared from connective tissue up to gelatine, the latter is the most degraded structure with the lowest stability to further degradation (Fig. 12).

To achieve materials with mixed properties e.g. higher stability to enzymatic degradation and also hemostyptic properties, gelatine may be combined with fibrous collagen preparations [221]. Combinations of gelatine gels and collagen can be prepared by utilizing the temperature gap of T_D_ and the setting temperature of gelatine which is usually lower than 30 °C. If the gelatine sets, two melting temperatures can be measured. One reflects melting of gelatine gels, the other T_D_ of collagen [238].

### Process steps affecting the mechanical stability

Biomaterials for clinical applications have to be easy to handle, should be able to be applied by surgical techniques, degradability and tissue integration have to follow the regeneration of the surrounding tissue. The key properties are mechanical stability and enzymatic degradability which are strongly influenced by mechanical disintegration, denaturation of the triple helix and crosslinking.

**Fig. 14:**
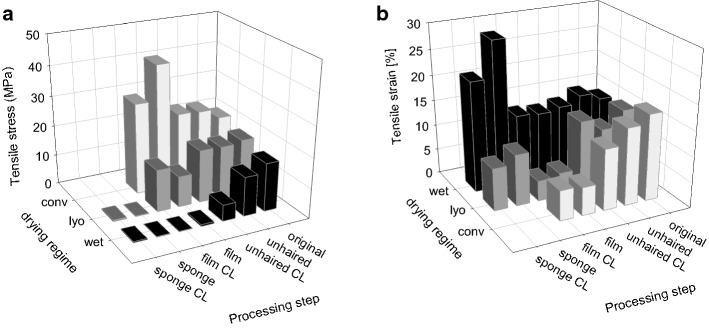
**a**, **b** Tensile stress and strain of porcine skin depends on the drying regime and physical processing (wet: no drying; lyo: freeze drying; conv: convection drying). Consider the inverted direction of the *drying regime* axis between both figures [209]

The mechanical properties of hide along with exemplary processing are compared in Fig. 14a, b. Hide samples were prepared with regard to different processing stages. Skin shows a tension at break of around 16 MPa and the mechanical properties were only slightly influenced by chemical purification processes. Unhairing was performed with intensive alkaline (pH 12.8) and reductive treatment (3% Na_2_S) for 16 h, and neutralized. Similar results were found by Damink et al. [239] for sheep skin. Porcine skins, which are split to a convenient thickness, are used for hernia repair or as temporary skin replacement e.g. Strattice^®^, XenMatrix^®^ or Xenoderm^®^ [240–242].

Mincing causes destruction of the fibre network. When unhaired hide tissue is disintegrated to a suspension, convection-dried as film and re-wetted for the measurement, the stability is decreased by around 90% in wet state, measured as tension at break compared to the tissue. Films manufactured this way are e.g. applied for hernia repair, temporary skin replacement or in dental applications (e.g. Resodont^®^ or Biomend^®^).

Dry films (Fig. 14a) show a much higher stability which is caused by the collapse of the swollen structure and agglutination of the fibres during convection drying. This agglutination is much less intensive when the suspension is not convection-but freeze-dried. In this case the mechanical stability is very low, leading to sponges which are used in dentistry as hemostyptic or as wound dressings e.g. Matristypt^®^, MBCollagen^®^ or others [1, 243–246]. These are not useful for load bearing applications. If films or sponges are dry, the higher stability improves surgical handling. In wet state the stability is rather low, however.

Crosslinking does not necessarily improve the mechanical stability of skin samples and collagen films in wet state (Fig. 14a; [247–250]), but the stability of sponges increases to some extent (Fig. 14a; [251]). The stability of the original tissue in wet state cannot be achieved again, neither by film preparation nor with sponges prepared from disintegrated tissue. The elongation values correspond inversely to the values for tension at break.

The parameter tension at break (UTS) is of only limited importance with regard to the clinical application, because it refers to the cross-section area of the samples. Processed materials may reach high values as summarized in Table 8. Because of the small thickness e.g. of films (approx. 20 to 100 micrometers) or the low diameter of threads (approx. 50 to 300 micrometers), the tension at break can become very high, though the force at break is low. In contrast, collagen sponges show very low values because of their porous structure and the resulting high thickness.

**Table 8:** Ultimate tensile strength (UTS) of collagen-rich tissues, decellularized, and further processed materials

Material	UTS (MPa)	Refs.
Raw material		
Tendon	30–300	[47, 497]
Ligament	30–300	
Aorta	0.3–1.7	
Skin	1–16	
Pericard	3–18	[498]
Decellularized		
SIS based	3–22	[499, 500]
Dermis based	10–39	[40, 252, 500, 501]
Pericard	8–32	[252, 500, 502]
Cast		
Thread	2–56	[265, 503]
Film	30–40	[250]
Sponge	0.001	[504]

**Fig. 15:**
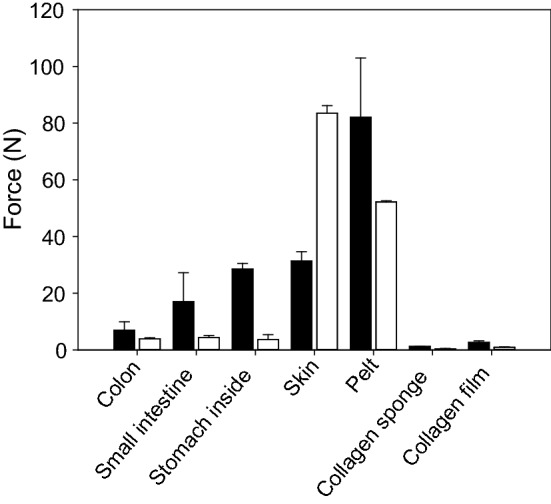
Ultimate tensile force (black bars) and stitch tear force (white bars) measured in wet state of different tissues, collagen films and sponges prepared from collagen suspension [209]

If the force at break and the stitch tear resistance of film and sponge are compared with real tissues Fig. 15), it becomes obvious that skin is very stable compared e.g. to intestine tissue and that films prepared from collagen suspensions show tensile strengths near the values achieved for less stable tissues like intestine. Therefore, force at break, burst strength, or stitch tear resistance better reflect the real surgical application than e.g. tensile strength as also discussed by Deeken et al. [242].

### Process-dependent variation of the enzymatic degradability

**Fig. 16:**
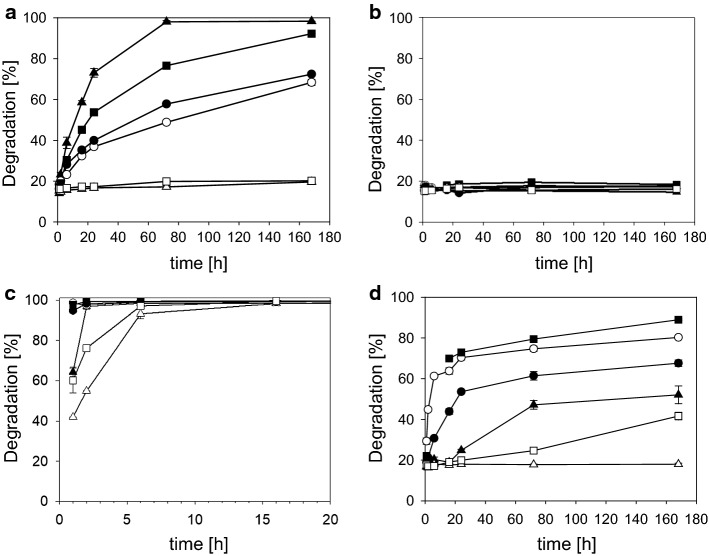
**a**, **b** Digestibility of porcine hide depending on treatment time, kind of enzyme without (left) and with synthetic crosslinking (right); filled triangle: collagenase A; open circle: proteinase K; filled square: pronase E; open triangle: trypsin; filled diamond: thermolysin; open square: chymotrypsin. All experiments with the same enzyme activity (unpublished data by Michaela Schroepfer, FILK). **c**, **d** Digestibility of heat-treated, denatured porcine hide depending on treatment time, kind of enzyme without (left) and with synthetic crosslinking (right); filled triangle: collagenase A; open circle: proteinase K; filled square: pronase E; open triangle: trypsin; filled diamond: thermolysin; open square: chymotrypsin. All experiments with the same enzyme activity (unpublished data by Michaela Schroepfer, FILK)

Triple helical collagen is stable to many proteases except collagenases. Additional crosslinking further stabilizes the collagen. Decellularized porcine dermis acts as effective substrate for bacterial collagenase, and with decreasing susceptibility for Pronase, Thermolysin and Proteinase K. Trypsin and Chymotrypsin are not able to digest the collagen structure in 180 h at 37 °C (Fig. 16a). Crosslinking with hexamethylendiisocyanate (HMDI) completely supresses the degradation by all tested enzymes (Fig. 16b), as well as that with low amounts of collagenase (unpublished own investigations; similar material to Permacol^®^). However, collagenase degrades crosslinked tissue at higher concentrations (sheep skin) [239]. The tensile strength and also the strain was found to decrease by enzymatic digestion down to a stable limit [239, 252].

The enzymatic degradability is relieved when the triple helices of collagen are denatured. Then, the protein chains are easily susceptible to the degradation by all mentioned enzymes within a very short period of time (Fig. 16c). If chemically crosslinked material is denatured by heat, it becomes degradable as well within similar time periods (Fig. 16d) as without denaturation and crosslinking. But, the order of the enzymes regarding their activity changes (Pronase; Proteinase K are more active than collagenase) and the degradation with any enzyme is complete within 180 h.

Obviously, crosslinking affects the mechanical properties in wet state and the degradation behaviour of collagen materials in vitro and in vivo. An implanted biomaterial causes an intensive cascade of interactions with the host tissue [28]. As first reaction (minutes to hours), hemostasis is observed and the formation of a fibrin-rich matrix, followed by an immune response of neutrophiles and M1 type macrophages. Cellular debris is degraded, pathogens are inactivated and the scaffold begins to degrade releasing matricryptic peptides (days up to weeks). M2 type macrophages invade the matrix and initiate remodelling of new tissue by a complex cell population (weeks up to months). Therefore, the degradation and remodelling behaviour of collagen is caused by a finely tuned equilibrium of M1 type and M2 type macrophages.

Hence, additional chemical crosslinking performed to prolong degradation in vivo is discussed controversially. From the clinical point of view, various authors [253–255] recommend to completely avoid chemical crosslinking because of the intensive proinflammatory reaction to crosslinked materials, the risk of scar formation, encapsulation and low vascularisation. In contrast, Dunn [256] argued in a recent review that long term clinical investigations are still missing, in vivo trials with animals often lack comparable conditions and the existing results are not coherent enough. Depending on the clinical application and the function of implants, crosslinking seems to be advantageous with regard to physical properties such as strength over time, the integration in the surrounding tissue and the rate and type of remodelling.

Rothamel et al. [254] compared the degradation of differently prepared biomembranes in vivo with and without crosslinking (Table 9). The samples were allocated in subcutaneous pouches on the back of rats. Porcine membranes, which were not additionally crosslinked, were integrated and vascularized after 2- to 4 weeks, while highly crosslinked tendon-based membrane was not fully integrated and vascularized during 24 weeks. In contrast, biodegradation was the lower the more intensive the collagen was chemically stabilized. The measured stability to enzymatic degradation in vitro could qualitatively be confirmed in vivo though the time scales are completely different. The degradation in vivo is observed to be much longer than in vitro at ideal conditions.

**Table 9:** Degradation in vivo of commercial products based on different tissue sources and differently crosslinked [254, 507]

	Supplier	Crosslinking	Species	Degradation in vivo (weeks)
BioGide	Geistlich Biomaterials		Porcine skin	2–4
BioMend	Sulzer Medica; now Zimmer Biomet	Glutaraldehyde	Bovine tendon	4–8
BioMendExtend	Sulzer Medica; now Zimmer Biomet	Glutaraldehyde	Bovine tendon	18
TutoDent	Tutogen	–	Bovine pericard	8–16
Ossix	Colbar R&D ltd.; now datumdental	Enzymatic/carbohydrate	Bovine tendon	> 24

### Shaping

To manufacture implants with different complexity for various clinical applications, three strategies can be refined to achieve the final goal of clinical success.

1. Supply of simple structures as already marketed e.g. membrane or sponge. Decellularization of these structures has been performed for many years with tissue parts as e.g. skin, cartilage, bone, pericardium or intestine. The techniques are established and led to many marketed products [3, 20, 257–259], which are used by surgeons in low vascularised areas e.g. for skin, cartilage or bone replacement, for dental applications or when only membranous structures are required. Usually the surgeon adapts the simple structures of the biomaterial as sponge or membrane to the clinical need and the surrounding recipient tissue revascularizes and metabolizes the implanted material.
2. Supply of complex structures as decellularized organs containing saved vessel trees which are then recellularized in vitro. For two decades decellularisation of whole organs provided impressive results, as cell free scaffolds of whole organs as heart, liver, lung, brain, kidney and others. These investigations aimed to save the complex structures of the organs, especially the vascular networks and the mechanical stability of the constructs [18, 23, 214, 218, 260, 261] and others. However, a recent paper describes the obvious drawbacks of the decellularization of the whole organs [28]. It is very difficult to control the final purity of the structures and the complex structures of blood vessels and their capillaries remain a challenge to be recellularized.
3. Hybrid structures between these two are basing on purified intermediates. From the engineering point of view and with the aim to manufacture such hybrid scaffolds or tissue components, tissues can be reduced to three basic shapes [209]. These are found in different parts of the body: voluminous porous structures as liver, kidney, lung, bone, cartilage, but also muscles and skin in the broadest sense; membranous structures as pericard, peritoneum, urinary bladder, parts of the skin (e.g. epidermis), intestine; and finally tubelike structures as blood vessels in their varying dimensions or nerves. This view is of course a simplification and a question of magnification. E.g. tissue of intestine in microscopic view appears like a membrane and has to be assigned as a tube in its complete cross section.

The latter strategy requires different shaping techniques. Tissue based cell free intermediates which are further disintegrated into freely shapeable pastes, gels, sheets, or powders [7, 27] can be formed into diverse parts as films, tubes, porous foams, and complex materials of different porosities, membrane-like structures, which cover sponges that contain hollow-fibres. The techniques of shaping comprise extrusion, mould casting, injection moulding, textile techniques, electrospinning, and end up with additive manufacturing techniques which allow to manufacture very complex structures [13, 209, 222, 262]. This strategy allows great latitude with regard to size, design and material, but it lacks the redesign of complex shapes such as vessel trees or plexus. It can be presumed that complex structures will be rebuilt by biological means in vitro or in vivo by cellular seeding [263].

#### Extrusion

Extrusion means pressing out of highly viscous masses through a die. The technique is widely used in the plastics industry to form synthetic thermoplastic polymers into strands with complex designs. The die can be just a hole resulting in cylindrical strands, a flat die leading to films or a ring-shaped die to manufacture tubes.

In case of extrusion of collagen suspensions through dies with rotating installations in cold state, collagen fibres are oriented in specific angles leading to higher mechanical stability in various directions than by extrusion without this adjustment [2, 222]. Tube extrusion of collagen masses in cold state had first been established several decades ago to manufacture sausage casings [219]. Collagen suspensions have also been extruded to manufacture threads and filaments [13, 232, 264, 265].

By hot extrusion it was also possible to process thermoplastic collagen [232], and extrusion in cold and hot state may also be a shaping technology for additive manufacturing [13, 266, 267].

#### Casting from moulds

Moulds, which have been manufactured by subtractive machining or by stamps, are used to prepare shaped articles. The latter technique is used to manufacture wine gums. A highly viscous gelatine solution containing other additives, such as e.g. aroma, sugar and citric acid, is poured into moulds prepared by a starch powder bed similar to sand casting in the metal industry. The positive mould is manufactured by cutting from a gypsum block. It is used permanently, the negative mould only one time.

Permanent moulds are used to prepare sponge-like structures in different shapes from collagen suspensions. The viscous preparations are poured into the holes and freeze-dried. The moulds are mostly made of metal to speed up the freezing process before drying. Cones and cylinders are produced for dentistry (Parasorb cone^®^), and cube-like sponges from gelatine (Gelita-Spon^®^).

Moulds have also been made by additive manufacturing [268] or by handcrafting to achieve more complex shapes e.g. an auricle or a bladder-like structure [269, 270].

#### Injection moulding

Injection moulding describes an economized technology to fabricate 3D articles by injecting a hot melt of thermoplastic polymer into a mould. This mould is then cooled down and the solidified article is rejected. Clock speed is seconds up to minutes depending on the size of the article and the cooling regime. Though collagen is initially not thermoplastic, the thermoplastic intermediate can be shaped by this technique [205, 230]. The partial denaturation, subsequent grinding and extrusion allowed to manufacture articles from thermoplastic collagen e.g. small shapes such as pet food but as well hard articles. The recipes only contain partly denatured collagen, water and glycerol as plasticizer. The preparations could also be mixed with hydroxyapatite to manufacture bone-like structures for medical applications. After drying and without addition of glycerol, the articles became bone-like and very strong.

#### Textile techniques

In the past, many trials were performed to transfer collagen into threads or filaments. Catgut had been used for centuries as surgical sewing thread and Franz Kuhn was the first who was able to completely disinfect the material by proving all manufacturing steps according to their purity [271]. Catgut was manufactured from stripes of the gut of goat or sheep and other mammals, which were purified mechanically, decellularized, limed, twisted until a cylindrical shape was achieved and dried under tension. To adjust the degradation rate in the body the strings were also crosslinked with chromium salts. The same material was used as strings for music instruments, and by surgeons to close wounds. By glueing several stripes together, it was possible to manufacture threads with sufficient yardage.

Catgut is completely degradable in the body and shows high strength and a high elasticity. However, it disappeared from the medical market because of the risk of bovine spongiform encephalopathy (BSE) and because synthetic alternatives came up [272–274].

Though collagen is a fibrous protein it is not possible to manufacture long fibres by direct spinning like it is common with other natural fibres such as wool, silk or cellulose. Collagen neither grows as filamentous material (wool, silk) nor can it be solved in full substance to be spun from highly concentrated solutions.

Nevertheless, there are three ways to produce fibres of high yardage [232, 265] (and the cited literature therein). Firstly, it is possible to solvent spin collagen from low concentrated solution and precipitate a fibre in organic solvents or aqueous salt solutions. Crosslinking by glutaraldehyde, EDC or isocyanates leads to more or less stable fibres.

A second way is to use collagen suspensions from decellularized minced hides or tendon which are extruded as fibre. The principle had been established many decades ago to manufacture sausage casings but can easily adjusted from tubes (casing) to cylinders (thread). The challenge is to chop up the collagen raw material sufficiently so that the swollen particles and fibres pass through the spinning nozzles. The spun fibres have to be precipitated and crosslinked as solvent spun fibres.

The third technique is melt extrusion of thermoplastic collagen. Here the primary fibrous structure of the collagen has been lost because the collagen is denatured and melt spinning is performed similar to the spinning of thermoplastic synthetic polymers. The resulting collagen filaments are highly stable in dry state, but they are very sensitive to humidity and lose their stability when humidified.

It is possible to knit and weave such filaments, but embroidering and sewing requires very high stabilities of the threads when passing the eye of the needles and thread breakage is observed very often. Though all three technologies allow to manufacture textiles from these filaments the wet stability of all collagen textiles is not as high as it can be achieved with other filaments [232, 275].

#### Electrospinning

Electrospinning is a technique to manufacture nanofibrous non wovens by ejecting polymer solutions or melts through a die in a strong electric field. The leaving droplets are deformed and build filaments which are deposited on a support. The solvent has to be removed during the transfer of the filament from the die to the support. Collagen solutions had already been applied using 1,1,1,3,3,3-hexafluoro-2-propanol as solvent [276–278]. If collagen is spun from aqueous solution, it usually denatures [279]. Only scarce investigations successfully exchanged the toxic fluorinated solvents with nontoxic ones while saving the triple helical structure. The authors used mixtures of aqueous ethanol, sodium chloride or polyvinyl alcohols and first applications in the medical field e.g. guided bone replacement are in preparation [280–282]. Electrospinning only works from solutions. Collagen suspensions could not be spun this way, because the viscosity is too high to be extruded as thin filament, and water or aqueous acids as solvent could not be evaporated sufficiently to prevent agglutination (own unpublished results).

#### Additive manufacturing

Additive manufacturing (AM) comprises a rapidly growing number of techniques to construct 3D articles not by cutting and machining (substracting techniques) or by moulding but by additive techniques. Though metals and ceramics are processed as well, polymers are the most intensively utilized materials class [283]. AM machines require CAD-based data sets which can be generated completely in silico or based on imaging procedures (e.g. X-ray, MRT, surface scans). These virtual models have to be formatted for AM to be realized in form of layer by layer techniques. AM techniques cover photopolymerization (stereolithography), powder bed fusion (SLS), material and binder jetting (inkjet and aerosol 3D printing), sheet lamination (LOM), extrusion (FDM, 3D dispensing, 3D fibre deposition, and 3D plotting), and 3D bioprinting. The technological principles and possible materials have been recently summarized in a high number of publications focusing on the principles [284], on synthetic materials as metals, ceramics and synthetic polymers [285], more comprehensively on machines, processes and materials, including bioinks [283, 286–289], and finally on the manufacturing of parts or whole organs [290–295], to cite just a selection of the last years.

When focusing on solely processed collagen and its derivative gelatine without other polymers, much less has been published yet, though both materials are highly suitable from the biological aspect [296]. Drawback is the collagens ambitious processing and its specific thermal behaviour. Thermoplastic collagen does not act really thermoplastically which would be necessary for extrusion. In contrast to synthetic thermoplastic polymers, which melt at higher temperatures to achieve low viscosities, thermoplastic collagen behaves gum-like and has to be extruded under shear [205, 230]. For this purpose, suitable plotting machines have not been developed yet. Gelatine solutions show very low viscosity as warm solution. Setting is possible but it is too slow (minutes up to hours) to achieve suitable processing times. Solutions of triple helical collagen show low viscosity in cold state and heating should be avoided.

Panwar and Tan [297] summarized the different approaches to overcome these processing difficulties. Gelatine had been methacrylated to achieve a higher stability in wet state and to decrease the degradation rate [292, 298–300]. Furthermore, gelatine was combined with other biopolymers such as hyaluronic acid [300, 301], alginate [302], fibrin [303], chitosan [304] and silk [305] but also polyethyleneglycol [306]. These mixtures aimed to increase viscosity so that strands can be deposited. Alginate was solidified by deposition of gelatine alginate mixtures in a water batch which contained Ca^2+^-ions.

Collagen was first used as reassembled gel from collagen solutions combined with polyethylene–polypropylene–copolymers [307] and with many of the cited other biopolymers, some of them also being modified with acrylic groups to allow photo-crosslinking [297, 308–310]. Kim et al. [311] solidified extruded atelocollagen dissolved in acetic acid (dry matter content 4.5%) by depositing the solution on a plate which was cooled at − 40 °C. The frozen scaffold was then freeze-dried without thawing, and crosslinked after lyophilisation with EDC in ethanol.

Pati et al. [312] used decellularized porcine heart and cartilage which was physically homogenized in cold state, freeze-dried, pepsinised, filtrated and neutralized. The resulting suspension could be combined with cells in cold state. By increasing the temperature the bioink set, but to achieve stable structures the bioink had to be combined with polycaprolactone strands.

Jose et al. [313] formulated the requirements for an advanced bioink. It should be biocompatible and biodegradable over long periods of time, act as scaffold, and balance biological and structural properties. To achieve these requirements they proposed a combined deposition of structural and biological components.

In own trials, collagen and gelatine were co-deposited to manufacture biocompatible structures [267]. Collagen suspensions were prepared by mechanical mincing of decellularized porcine skin split in cold state which was then sterilized by peracetic acid. To achieve cytocompatible preparations the acidic masses were adjusted to neutral pH by addition of sodium hydroxide. Precipitation of the fibres was suppressed by addition of 250 mmol/L TrisHCl (pH 8). These preparations could be directly plotted with a bioscaffolder (GESIM, Radeberg, Germany) by wet extrusion. A fibroblast cell suspension was applied as bioink which consisted of a warm (20 °C) gelatine solution (2%) in PBS. Settling of the cells was prevented by addition of reassembled collagen in low amounts. This combination allowed to directly manufacture complex 3D structures with live cells in one process based only on collagen and collagen derivatives as structure forming polymeric component and without any other polymeric additives. In future this technique may be used to manufacture individual tissue-like structures.

### Specifications and limits

Processing of collagen requires target limits for the purity of the processed materials. For medical applications, which shall be marketed, these materials are often characterized according to standards e.g. ASTM F2212-11 and the cited ASTM, ISO and European standards therein. These standards summarize analytical methods but present only few limits.

It can be taken for granted that materials, which are clinically used, have to be free of any viable microbiological bacterial contamination [210]. This is achieved by adequate sterilization. With regard to collagen this can be a challenge and has to be planned at the beginning of a product development. Further, the endotoxin content is limited to 0.5 EU/mL extract and 20 EU/device according to governmental regulations (http://www.fda.gov) which can be technologically achieved as described below. Cytotoxicity is usually measured directly from the material or from an eluate e.g. using the XTT test. Prior to the test it is important to adjust pH and to ensure that no remnants of processing agents e.g. peractic acid, hydrogen peroxide, salts or sterilants lead to cytotoxic results.

The purity, that has to be achieved, comprises biological components which remain from the decellularized tissue, and remnants of organic and inorganic processing chemicals. Londono and Badylak [28] recommended an upper limit of 50 ng DNA/mg dry weight, no DNA fragments longer than 200 bp and no visible nuclei stained by Hämatoxylin/Eosin or DAPI as marker for sufficient purity. These limits are not a biological requirement, but they have been used to indicate sufficient chemical cleaning [314].

Fats and lipids are usually removed as much as possible and can be detected by extraction and chromatographic analysis. Remnants of inorganic components are easy to be measured (AAS, REM-EDX, IC, others). However, highly effective organic additives, especially surfactants and applied enzymes, are difficult to detect, though ToF–SIMS seems to become a powerful tool for such analyses [172].

Other non-collagenous remnants are discussed controversially such as impurity, or as desired ingredients which have to be saved or removed during processing, e.g. bound carbohydrates, growth factors and elastin [27, 315]. However, it seems to be a challenge to selectively maintain specific components in reproducible quality during complex chemical manufacturing processes. Presumably, it is more effective to add selected additives after intensive purification as it has already been established for drug delivery systems.

Regarding functional properties it is reasonable to detect the nativity of collagen, because this can be directly correlated with many properties such as hemostypic behaviour, degradability and mechanical fastness. ΔH_D_ is the key parameter to be measured by DSC. Native soluble mammalian collagen shows ΔH_D_ of 50 to 60 J/g collagen [132, 316].

Crosslinking can be detected by amino acid analysis in combination with DSC and solubility measurements. By crosslinking T_D_ increase compared to non-crosslinked materials, selected amino acids disappear (e.g. Lysine) and the solubility and sensitivity to proteases decrease.

## Processing collagenous tissues—the tools and their effects

Processing of collagen-rich tissues to manufacture biomaterials follows some principles which had been developed over decades (Fig. 11). Usually, the aim is to liberate the tissue as consequently as possible from non structure forming components. The resulting materials are processed into different shapes and often finally dried and sterilized. Depending on the used technology, sterilization again affects structure and chemical properties, however. The following section will discuss the influence of several single steps on the final material properties.

### Mechanical treatment

Often it is convenient to remove unwanted parts of the targeted tissues by mechanical means. While this is performed for many small-sized preparations like pericardium, tendon or urinary bladder personnel-intensive by hand, automatic methods are available for intestine and skins, because the latter raw materials are used by the food and leather industry.

#### Hide fleshing and splitting

The mechanical steps fleshing and splitting are used to purify skins, remove unwanted structures and to adjust thickness. Fleshing removes the subcutis by shaving the fatty tissue with a rotating knife cylinder [317]. The skin is moved and removed from the machine by transport rolls. The skin is applied between a knife cylinder and a support roll and pressed against the latter. The rotating knife cylinder removes fat tissue and also muscle and loose connective tissue from the backside. Own trials showed that higher amounts of fat e.g. from porcine skins are often pressed into the reticula by the rolls which leads to higher fat contents inside the collagen structure. In this case it is advantageous to split the hides to remove the fat.

Splitting machines can be used to divide whole skins e.g. from bovine, porcine or equine sources into splits of 0.3 mm thickness over an area of up to 5 square metres. Two pairs of specific transport rolls, one made of rubber and the second arranged as assembly made of brass, transport the hides into the machine. Splitting is achieved by a rotating band knife which is continuously sharpened [317].

Alternatively, in clinics dermatomes are used by surgeons to prepare skin autotransplants. These dermatomes work as cutting knife with fixed or vibrating blade and can only be used to generate small stripes of skin.

#### Degumming of small intestine submucosa (SIS)

To prepare SIS, intestines from pigs or other mammals are provided from abattoirs and mechanically treated in a roll system combined with washing. This way the submucosa is widely freed from other components such as mucosa and muscular tissue. The mechanically liberated submucosa is then treated similar to skin with pH sweeps and other agents.

#### Mincing and homogenization

Collagenous tissues, especially skin and tendon, are homogenized into suspensions prior to being shaped into films or foams. The physical treatments mincing, milling and homogenization are key steps during preparation of these fibrous collagen suspensions. The procedures usually end up with a highly swollen collagen fibre mass, which shows viscosities from flowable to stiff like a tennis ball, with dry matter contents between only 0.5 to 10%. They show shear thinning behaviour and their viscosity depends on pH, dry matter content, and the intensity of mechanical milling. By adjusting the pH higher than 5.5, the collagen fibres precipitate and water separates [200, 267].

Usually, the preparation of fibrous masses aims to save the triple helical structure. Therefore, it is crucial to prevent the material in every production stage from temperatures (also locally) higher than T_D_. As the swelling degree T_D_ is highly pH dependent, mincing and especially homogenization steps have to be finely tuned. Machines require powerful cooling techniques which are known from the meat industry. Meat choppers are used for coarse grinding in the first step when the raw material has already been chemically purified. Industrially applied technologies of homogenization e.g. used in the casings industry are divided into “dry” and “wet” processes. “Dry” means dry matter contents of 10 to 15% in an aqueous system with some additives. The “wet” process usually works with dry matter contents lower than 5% [318].

While homogenization during the “wet” process is achieved with colloid mills, during the “dry” process the preconditioned mass of highly swollen fibres is homogenized through a series of punched discs with very high pressures of several hundred bars. The resulting masses differ especially regarding their fibre lengths. The “dry” processed fibres are thicker and can be several centimetres long. When the masses are homogenized in a colloid mill, several hundred micrometres are achieved [219, 318]. The fibre distributions vary considerably and reach a very wide range in both process variants.

Real dry grinding (dry matter content > 85%) is also possible from dried tissues [27, 319]. This leads to wadding-like voluminous intermediates that can be re-swelled in wet aqueous systems and are homogenized by similar techniques as described for the “wet” process.

Recently, Terzi et al. [320] described correlations between different commercial equine, tendon-derived fibrous collagen intermediate materials, structural information and biological properties of prepared films. Unfortunately, the preparation techniques of the fibrous intermediates have not been exactly presented. It is difficult to evaluate such results, because the processes, the technologies of fibre preparation steps and the preceding technologies markedly influence the final material properties.

### Physical treatment

Physical treatments such as extraction, temperature treatment and radiation affect the collagen on different structural levels.

#### Extraction

Nageotte [321] first discovered that triple helical collagen can be extracted from young tissues by organic acids, and to this day the principle of the preparation of soluble collagen did not change very much. Collagenous tissue is only partly soluble in buffering solutions and in organic acid, and it is largely stable against proteolytic digestion except collagenases [322]. The susceptibility against acid and proteases decreases with increasing age [323] which is a result of specific and unspecific natural crosslinking of the tissue [77]. The solubility also strongly depends on the species. From skins of 6 months old calfs up to 10% collagen can be extracted while porcine skin of similar aged animals is almost insoluble (< 0.5%). The skin of fish is highly soluble [324–327], and more than 50% can be extracted in one step.

Atelocollagen is generated by treatment of collagen-rich raw materials (skin, tendon) with pepsin in acidic solution. The non-helical telopeptides are digested including the crosslinks in this region, only the triple helical molecule parts (e.g. human α1(I), Position 179–1218) become solved and the yield is higher than by extraction with weak acid [2]. The digestion of these telopeptides is relatively slow. To completely remove them, reaction times of 24 h and more are necessary. The success can be controlled analytically to some extent by amino acid analysis (Table 3), because tyrosine in the telopeptides of α1(I) is only found in the positions 165 and 167 (N-terminal) and 1215 and 1216 (C-terminal), and therefore outside of the triple helical region but not directly adjacent to the triple helical region. The α2 chain of human collagen I contains only one tyrosine in the triple helical part. If analytics achieves only one tyrosine, the telopeptides have to be at least partly digested.

Finally, treatment of the raw material with alkaline transfers the amides glutamine and asparagine into the corresponding acids glutamic acid and aspartic acid. This soluble collagen is called desamidocollagen and shows markedly lower IEPs compared to collagen without alkaline treatment and only reduced fibril forming power [328].

#### Temperature

When collagen is exposed to different temperatures, three ranges have to be considered: (1) lower than freezing temperature of water or buffer, (2) between thawing and T_D_ and (3) higher than T_D_.

Temperatures lower than the freezing temperature are important during freeze drying. About 35% m water/m dry matter are bound to collagen and are non-freezable. Bound water solidifies only at temperatures lower than − 60 °C [329–331]. This might be the reason why mechanical properties and the higher structures of collagen are only slightly affected by freezing [20]. In decellularization protocols often freezing and thawing cycles are used to effectively damage the cells by physical means [20, 332, 333]. Multiple cycles are more effective than only one. However, the temperatures at which freezing ends as well as freezing and thawing rates have not been published. It is expected that very high or very low freezing rates damage the cells more intensively [334, 335]. Presumably, not only the number of cycles but also the rates of freezing and thawing will influence the efficiency of decellularization.

**Fig. 17:**
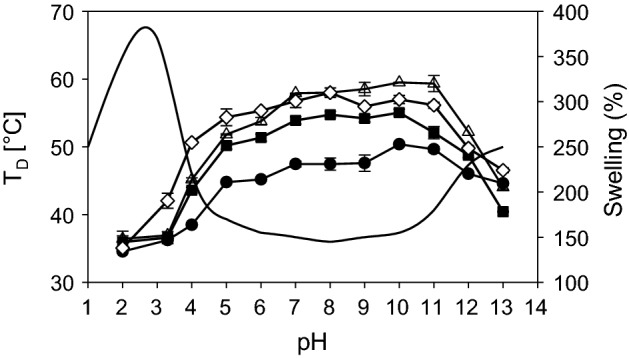
Denaturation temperature T_D_ [347] of different forms of collagen and swelling of sheep skin [343] depending on pH; T_D_: open triangle: rat tail tendon; filled circle: soluble collagen; filled square: porcine hide; open diamond: bovine hide; swelling: –

The temperature range between zero centigrade and T_D_ is the important process window when the triple helical structure is aimed to be saved during wet processing and shaping. All process steps have to be adjusted with regard to this range. This also concerns local temperature transgressions e.g. during mincing, milling or cutting steps. T_D_ not only depends on the dry matter content but also on the swelling degree (Fig. 17) which is pH-dependent. T_D_ may locally decrease because of the application of hydrotropica, acids or alkaline which are not homogenously distributed in the material. Therefore, a processing temperature sufficiently different to T_D_ has to be considered.

Passing T_D_ unlocks the triple helices. T_D_ depends on the water content of the fibrils which corresponds to structural organization (solved, fibrillar), the pH, but also the crosslinking degree [135, 155, 156, 181, 336]. In tissue, the fibrillar collagen structure under tension only partly undergoes a transition to a coiled structure. Soluble collagen molecules, which consist of single triple helices, disintegrate into the single protein chains which then adopt a coiled structure of one-third in length [337, 338]. In grown tissue, the fibrillar structure shrinks as much as fixing of the fibres by physical entanglements and chemical crosslinking allow the triple helices to shrink. This denatured collagen is highly susceptible to enzymatic degradation, and the mechanical stability decreases.

#### Radiation

Different kinds of radiation have been investigated in the past such as γ-, β-, and UV radiation. UV radiation is used as physical crosslinking technology, and it is more intensively discussed in the section of crosslinking. γ- and β-radiation are used to sterilize medical products. This is considered as well in the corresponding section.

### Chemical treatment

The chemical treatment aims to remove bound ions, carbohydrates, globular proteins, lipids, fats, nucleic acids and endotoxines from the tissue. Ideally, only purified connective tissue remains at the end without affecting the structure. Table 10 summarizes remnants and impurities of tissues to be treated, the used chemical additives to remove them and the reaction mechanism. Actions and effects on the collagen structure are discussed in more detail below.

**Table 10:** Impurities, agents to remove them and assumed reaction mechanisms

Unwanted adsorptive	Used agent	Removal mechanism
Monovalent cations	Acids (H+)	Ion exchange
Divalent cations	Acids (H+)	Ion exchange
	Chelating agents	Sequestration
Monovalent anions	Alkaline; chloride	Ion exchange
Saccharides	Alkaline treatment	Degradation
	Enzymes	Degradation
Proteins	Proteases	Degradation
	Solvent	Exchange
	Surfactants (Triton X 100; CHAPS)	Exchange and dissolving
Lipophilic components	Surfactants (SDS; Triton X 100)	Dissolving
	Solvent	Dissolving
Nucleic acids	Nucleases	Degradation
Endotoxines	Peroxide, alkaline, acid	Unknown

#### Acids, bases, pH and isoelectric point (IEP)

The isoelectric point (IEP) of collagen type I measured is 7.8 [339], though an IEP of 9.2 (a1(I)) and 9.9 (a2(I)) was calculated from the sequences (UniProt; bovine type I; P02453; P02465) of the triple helical part including the telopeptides using the ExPasy ProtParam tool. By treating collagen raw material with alkaline the IEP decreases. An intensive alkaline pretreatment is characteristic, when gelatine type B is manufactured, which leads to a partial desamidation of glutamine and asparagine to form glutamate and aspartate. An IEP of 5 or lower is then achieved [340, 341]. If all Gln and Asn are exchanged with Glu and Asp, the calculation with ProtParam leads to IEP 4.8 (α1(I)) and IEP 4.9 (α2(I)).

Collagenous tissue may swell by several hundred percent at a pH which deviates markedly from the IEP (Fig. 17). This swelling has been explained by the Donnan potential and by the rejection of charged protein chains which lead to an influx of surrounding water into the tissue [342]. The swelling has several consequences regarding the processing of collagen. The collagen molecules are separated and uncoupled from the stabilizing surrounding collagen molecules [135, 343, 344], and the material appears glassy and partly transparent. While soluble collagen is extracted, acidic pH creates a high internal tension of crosslinked collagen in tissue with a maximum of pH 3.

The separation of the collagen molecules leads to a decrease of the denaturation temperature by around 20 °C which is the difference between assembled and molecular collagen triple helices. Therefore, at pH deviating from the IEP the thermal stability of collagen in tissue is similar to soluble collagen, though it is not soluble, because it is still naturally crosslinked. The increased T_D_ in Fig. 17 for soluble collagen around neutral pH is a result of fibrillation or precipitation during measurement.

#### Hypo- and hypertonic treatment—the influence of salts

Ions have a multitude of effects on collagen depending on their charge, concentration and depending on the structural level of collagen tissues. To process collagen, it is of utmost importance to know how different salts affect the collagen structure. Some effects can be explained with the Hofmeister series, but especially the effect of multivalent ions and the effects on the structure of the fibril is complex.

Low yields of non-crosslinked collagen can be extracted at the IEP from tissue by isotonic solutions of neutral salts such as NaCl or sodium phosphate. Such collagen has been assembled in phosphate solution at 30–37 °C [66]. Under neutral conditions, univalent anions slightly stabilize soluble collagen at concentrations lower than 20 mmol/L which leads to an increase in T_D_. This is explained with charge screening. Between 20 and 500 mmol/L, different salts cause slightly lower or higher T_D_ depending on the position of the ion in the Hofmeister series (H_2_PO_4_^−^>SO_4_^2−^>Cl^−^>SCN^−^). Phosphate stabilizes while rhodanide reduces T_D_. Concentrations higher than 500 mM/L have been found to further increase T_D_ [316, 345, 346].

Furthermore, monovalent salts allow to suppress swelling at acidic pH by these charge screening effects. By addition of 2 mol/L NaCl, the swelling peak at pH 3 disappears almost completely, while the alkaline swelling remains unaffected [343]. This principle is widely used to prepare skin collagen for crosslinking while preventing pH-dependent tensions of the tissue. It becomes important during decellularization, because DNA is highly susceptible to a cleavage by acidic pH.

In tissue, several effects superimpose at the IEP. The observed stabilization or destabilization of collagen triple helix by ions, which are interpreted as salting in and salting out, competes with stabilizing and destabilizing effects of the assembled collagen fibrils. The different structural components may be separated by evaluation of T_D_ and ΔH_D_. While ΔH_D_ is a direct measurement of the triple helix stability, T_D_ reflects also an entropic part which correlates with the fibril stability. It is therefore possible to uncouple the ion effects on the different structural levels of collagen.

**Fig. 18:**
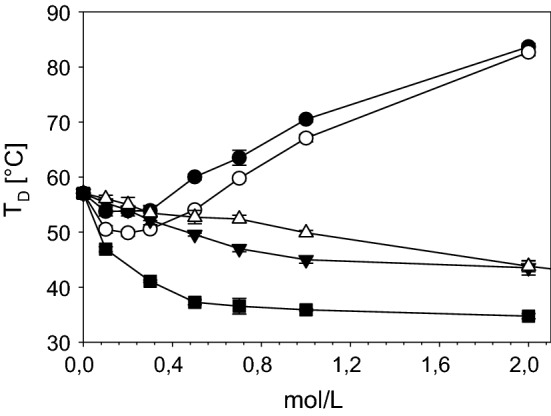
Denaturation temperature (T_D_) of porcine hide depending on different additive concentrations and different kinds of additives; filled circle: potassium phosphate; open circle: ammonium sulfate; down pointing filled triangle: tris–HCl pH7; up pointing open triangle: urea; filled square: calcium chloride [347]

**Fig. 19:**
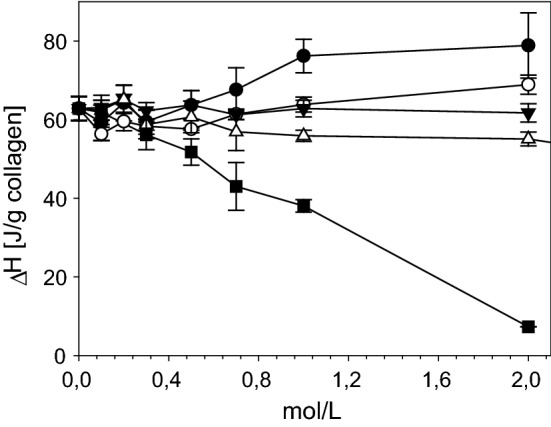
Denaturation temperature (T_D_) of porcine hide depending on different additive concentrations and different kinds of additives; filled circle: potassium phosphate; open circle: ammonium sulfate; down pointing filled triangle: tris–HCl pH7; up pointing open triangle: urea; filled square: calcium chloride [347]

Own results (Figs. 18, 19) [347] show the influence of different salts on T_D_ and ΔH_D_ of unhaired porcine hide collagen. After chemical hair removal, the tissue was adjusted to neutral, soaked in different concentrations of ions, and measured calorimetrically. The anions H_2_PO_4_^−^ and SO_4_^2−^ considerably stabilize the collagen fibrils. Between 0.5 mol/L and 2 mol/L T_D_ increases linearly by more than 20 °C while ΔH_D_ remains widely unaffected. In contrast, calciumchloride destabilizes the fibrillar as well as the triple helical structure, because T_D_ and ΔH are decreasing. Similar results were also found by Lim et al. [348] with bovine Achilles tendon.

Up to 2 mol/L urea and Tris(hydroxymethyl)-aminomethan buffered with diluted hydrochloric acid (TrisHCl) behave differently compared to other ions. At these concentrations, both only affect the assembled fibrillar structure leading to a decrease in T_D_ but do not influence ΔH_D_. The triple helical structure is not affected, but the fibrillary structure is partly destabilized at neutral pH. This is an important property when producing pH-neutral cell culture compatible wet collagen preparations.

#### Organic solvents

Ethanol, methanol, acetone and tributylphosphate are organic solvents which are commonly used to remove cell components, fat, lipids as well as endotoxins. Glycerol, ethyleneglycole and polyethyleneglycole are used as humidifying agents and as plasticiser of collagen materials.

Solvents affect the water shell around the collagen molecules. The addition of simple alcohols leads to unswelling as well as an increase of the denaturation temperature in tissue, and solved collagen is precipitated [349]. The use of polyhydric alcohols additionally stabilize the collagen against heat [350–352], but in the presence of KCl adverse effects are observed [353].

The use of organic flammable solvents leads to several drawbacks with regard to processing. They have to be removed completely, the plants have to be constructed according to requirements for fire protection, and it is not easy to remove the solvents by lyophilisation. Therefore, the use of solvents is tried to be circumvented and water-based systems are commonly used.

For some crosslinking reactions, especially with compact materials, it is advantageous to avoid aqueous buffers as solvent and organic solvents have to be used [265, 354–356]. E.g. hexamethyldiisocyanate (HMDI) is applied in dried acetone or 2-propanole, and EDC is commonly solved in ethanol. Otherwise, side reactions as the formation of polyurea (HMDI) or hydrolysis (EDC) may prevail.

#### Detergents

Detergents are primarily used to remove fats and lipids. Sodiumdodecylsulfate (SDS) or sodiumdodecylbenzene (SDB) and others are often used as anionic detergents [22, 123, 357–359]. Nonionic detergents usually belong to the Triton X series. The detergents not only liberate hydrophobic substances as well as GAGs, growth factors and non-collagenous proteins, but also adsorb to the collagen matrix [358, 360]. In certain cases, this may lead to swelling of the structure, but denaturation does not occur directly. A recent investigation showed that remnants of surfactants as well as other substances are found in decelluarized tissue also after intensive washing [172]. Because remnants of detergents are discussed to cause cytotoxic effects, recent technologies aim to avoid their use [361–363].

#### Chelating agents

Chelating agents bind polyvalent metal ions such as calcium, iron, cobalt and others. Typical decalcifying technologies e.g. of bone comprise intensive treatment of samples with e.g. ethylenaminotetraacetate (EDTA) to remove mineral components [364, 365]. EDTA is also used as agent in decellularization procedures. The exact impact is unclear, however [20]. Collagen as well binds polyvalent cations, and chelating agents compete with the collagen for these ions. Collagen is stabilized with iron, aluminium or chromium (Catgut) by the formation of complex bonds. By EDTA these ions can be completely removed. Therefore, the chelating capacity of EDTA is higher than that of collagen. The chelating agents neither affect the collagen structure nor the amino acid composition [366], but they have to be removed completely, because during formation of collagen by cells in vitro and in vivo the new collagen has to be hydroxylated by prolylhydroxylases which contain Fe^2+^ as central ion [367]. The action of these hydrolases is disturbed by chelating agents.

#### Reductive treatment

For more than one century it has been known that reductive agents are be able to dissolve keratin [368]. Reductive agents remove hair from porcine, bovine or equine skins without affecting the collagen structure. Today, usually sodium sulfide is used as reductive agent, but thioglycolic acid is possible as well. The reductive agents cleave the sulfur double bonds of cystine into cysteine and the keratin structure is destabilized. The reductive step is combined with alkaline treatment that cleaves the main chains and the keratin becomes solubilized. More recent own investigations show that also combinations of an alkaline treatment with proteolytic enzymes and homocysteine allow to solubilize keratin without affecting the collagen matrix [369].

The use of further reductive agents, especially to investigate chemical or structural effects on collagen, is limited to analytical approaches [87, 370, 371].

#### Oxidative treatment

In technical processes, collagen-based tissues are often treated with oxidative agents, especially hydrogenperoxide, peraceticacid (PAA) and sodium hypochlorite. Methionine is transferred into methionine sulfoxide or methionine sulfone, hypochlorite can lead to desamidation as well, and crosslinking may occur by generation of dityrosine [372, 373].

Hydrogen peroxide was observed in own trials to oxidize isolated and collagen-bound Met (1% H_2_O_2_ over night at room temperature; unpublished), compared to other observations which did not observe an oxidation of Met of other proteins [372]. Dityrosine in collagen was found to be formed only in the presence of tyrosinase and hydrogenperoxide [374, 375].

Regarding collagen processing technologies and subsequent material analyses, the oxidation of Met in collagen into its sulfoxide has two consequences. Firstly, the commonly used treatment with BrCN, which cleaves the collagen at Met and produces a small number of peptides with exact molecular weights, does not work anymore. BrCN-cleavage and separation of the peptides is one important technique to distinguish the collagen types [376–379]. Therefore, this analytical technique does not lead to results with oxidatively treated collagen.

Secondly however, Met can be used as efficient oxidation marker in collagen structures. If collagen materials are sterilized by peracetic acid, it is possible to prove the efficient treatment by the decrease of Met through amino acid analysis. The number of Met in skin collagen has been found to reduce from 8 to 2 or 3 after 1 h treatment.

#### Enzymes

More than one century ago, Röhm [380] invented the use of industrially produced pancreatic proteases to remove non-collagenous proteins from skins. Today, the use of many different degrading enzymes is established, such as specifically or broadly acting proteases, DNAses, lipases and carbohydratases, albeit the use of proteases is the most important. In some applications, enzymes may also be applied as crosslinking agents.

#### Proteases

The triple helical structure of collagen is very stable against enzymatic degradation. Collagen in vivo is almost exclusively digested by matrix metallo proteinases (MMPs), a group of endopeptidases which specifically metabolize connective tissue proteins [381]. In bone and cartilage the collagen can also be cleaved by cathepsin K [382]. Vertebral MMPs split the triple helical collagen type I into ¼ to ¾ at exactly one position between Gly 775 and Ile 776 [322]. Most MMPs consist of a pexin part which is able to recognize the cleavage site and unwind the collagen triple helix, and a catalytic part which cleaves the collagen main chains. Therefore, MMPs are responsible for the fragile equilibrium of collagen turnover in vivo [383–386]. Dispase is a special bacterial collagenase which mainly affects fibronectin and collagen IV and only to a minimum extent collagen I. It has been used to remove intact epithelial sheets and epidermis from their substrates [387], but it was necessary to combine it with trypsin to remove all cellular components to effectively decellularize a porcine dermis [388].

In contrast, non-collagen specific proteases only affect the non-helical parts (telopeptides) of the collagen chains leading to isolated triple helical molecules [389]. This is used technically to increase the yield when soluble collagen is manufactured. The collagen of young animals is primarily crosslinked in the telopeptide region. Therefore, cleavage of the telopeptides increases the solubility. Only the triple helical part of collagen type III is affected by more unspecific serine proteases.

As it cannot be excluded that remnants of enzymes remain in the purified material, the use of enzymes should be reduced to a minimum when preparing collagen-based biomaterials and decellularization technologies [362]. Nevertheless, many technological protocols use an enzymatic step in their processes. Then, it has to be ascertained by suitable techniques (e.g. ELISA) that the processing enzymes have been completely removed.

#### Other degrading enzymes

Other enzymes have been discussed to hydrolyse specifically non-proteinaceous components such as carbohydrates, proteoglycans, nucleic acids, and some lipids.

Lipases cleave ester bonds of triglycerides and cholesterolesters and their corresponding fatty acids, but solvents and detergents are more effective to remove lipophilic components [20, 390].

The most important group of enzymes, which are used beside proteases in decellularization, are nucleases to remove cellular remnants of RNA and DNA [390–398]. DNA remnants have been found in many final products [314, 399], but it is not clear whether DNA itself causes adverse effects or DNA only acts as marker for cellular remnants [400]. Own trials showed that especially acids (< pH2) effectively attack DNA, but also that adsorbed DNA is much more stable against deterioration [401].

The use of nucleases seems not to be necessary, because nuclease-free protocols are available which as well lead to sufficiently low DNA levels [390, 393]. As a purity marker for a successful decellularization, Crapo et al. [20] proposed a maximum of 50 ppm DNA with lengths not more than 200 bp and no visible nuclei after DAPI and hematoxylin and eosin staining as indicators for sufficient decellularization.

#### Transglutaminases

Enzymes, which catalyse the transfer of an acyl group between protein-bound glutamine and the ε-amino groups of lysine leading to ε-(γ-glutamyl)lysine isopeptides, are named transglutaminases (Tgase) [402]. Therefore, Tgases are able to crosslink proteins and to act as biological glue. They are widely spread in the animate world from microorganisms up to mammals and plants [403]. Tgases have also been used to stabilize collagen and denatured collagen (gelatine). It was found that porcine Tgase was able to slightly increase T_D_ of collagen up to 66 °C which argues for crosslinking of the triple helices [404]. In contrast to mammalian Tgase, the microbial variant was not able to stabilize triple helical collagen but only the denatured form gelatine. It was found that the reaction directly depends on the denaturation degree of the triple helices. Tgase adsorbs intensively to collagen matrices and it was demonstrated that in spite of washing with an excess of water four times, half of the Tgase still remained in the material [405, 406].

### Removal of endotoxins

Endotoxins are lipopolysaccharides of the outer cell membrane of Gram negative bacteria. They are composed of a core region which consists of oligosaccharides linked to the cell membrane-based lipids, and a highly varying polysaccharide chain which represents the antigenic part (O-antigen). Endotoxins are also liberated after the death of the bacteria and their removal is a challenge, because they are stable against heat up to 180 °C, solvents and many other chemical agents. Raw material to manufacture biomaterials as e.g. skins, tendons, intestine and other tissues are already contaminated by sourcing, and also containers, tubes, pumps, and chemicals may contain endotoxins, but the most important source for contamination is the laboratory water [178].

To remove endotoxin contents from thermal sensitive biological materials, it was found that a treatment with strong acids (1 N HCl), alkaline (1 N NaOH) and ethanol 70% may sufficiently reduce the contamination [178, 407]. Own trials with insoluble collagen materials showed that even the analytics depends on an accurate sample preparation, and that it is necessary to decompose the material accurately before testing. To remove the endotoxins, the alternating use of extreme pH changes (pH 2; pH 14) in cold state and the application of hydrogen peroxide was successful [179, 180].

### Special case—synthetic crosslinking

Synthetic crosslinking stabilizes collagen against temperature, enzymatic degradation and mechanical load in wet state beyond its stability achieved by reassembly and natural crosslinking. The literature on crosslinking methods is comprehensive because of the long tradition to make leather as chemically stabilized collagen material. To stabilize biomaterials, crosslinking can be achieved chemically and physically (for review see [408]).

#### Chemical crosslinking

Chemicals are used to stabilize the collagen for technical, medical and pharmaceutical applications. The side chains of the amino acids of collagen allow many different chemical reactions. The methods using polyvalent cations, which had been used also for medical products in the past e.g. stabilization of catgut by chromium ions, will not be considered, because it is not relevant anymore for biomedical applications. Other agents are bifunctional aldehydes, isocyanates, carbodiimides and acylacides, epoxides and some natural agents extracted from plant parts.

Under wet conditions, the temperature must not exceed T_D_ when the triple helix shall be saved. This requires ambient environmental conditions at temperatures lower than 60 °C. The use of solvents is technologically more expensive, needs to follow additional safety aspects and requires to discuss effects which result from excessive drying. The use of solvent, however, allows to use higher processing temperatures, and porous structures (sponges) are prevented from collapsing caused by capillary forces.

#### Aldehydes

Primary aldehydes react with ε-amino groups of lysine. The most common are formaldehyde and glutaraldehyde, but both of them react differently and several reaction channels are discussed [409, 410]. The reaction of formaldehyde can be easily achieved in gas phase or by soaking in dilute solution under neutral conditions. The reaction is reversible, and formaldehyde liberates from the treated material again e.g. by heating. Nevertheless, gelatine sponges to be used in surgery as hemostyptic are stabilized this way.

Crosslinking with glutaraldehyde leads to a markedly increased T_D_ and to high stability against enzymatic degradation. Materials are mostly processed by soaking in solution, but a successful treatment in gas phase is also possible at room temperature. A couple of different reaction channels have been discussed with intermediates that react further to become complex structures, which has been summarized by Damink et al. [249]. At the end, each amino group on average reacts with three glutaraldehyde molecules. In the past, the use of glutaraldehyde to stabilize biomaterials was intensively discussed. The aldehyde may be liberated again during degradation and could cause toxic effects [411, 412], and crosslinking with glutaraldehyde initiates calcification [413–415] though the exact mechanism is not clear. Zilla et al. [416] found that a very high concentration of glutaraldehyde can prevent calcification. Others showed that the treatment of glutaraldehyde crosslinked pericardium with glycine to prepare cardial valve leaflets prevented calcification [417]. Other bifunctional aldehydes are as well able to stabilize collagen, e.g. acrolein, glyoxal, malondialdehyde, succinaldehyde and dialdehyde starch [410]. They also cause different risks and the exact reaction mechanisms are as well often not known. Therefore, an intensive search for alternative crosslinking agents had been performed during the last years.

#### Isocyanates

Collagen can also be crosslinked with bifunctional isocyanates of which hexamethylene diisocyanate (HMDI) is the most common. HMDI reacts with ε-amino groups at room temperature. It is only sparingly soluble in water and needs to be emulsified by the use of surfactants [247]. At neutral and higher pH the reaction is faster than at acidic pH. If the reaction is performed in water, highly insoluble poly-urea is formed as byproduct. Therefore, crosslinking of thicker collagenous tissue such as skin (Permacol^®^), which requires suitable diffusion of HMDI into the tissue prior to its reaction, is usually performed in solvent e.g. 2-propanol or acetone [354]. Water-based systems or DMSO were used to stabilize pericardium by polyurethane prepolymers which still possess reactive isocyanate groups [354, 418].

#### Carbodiimides and acylazides

Both, carbodiimides and acylazides react with the carboxylic groups of collagen. The most common used carbodiimid ethyl-3(3-dimethylamino)propylcarbodiimid (EDC) initially forms O-acylurea groups with carboxylic side chains which then react with ε-amino groups to form isopeptide bonds. Beyond this new peptide group, no new bond or group is incorporated, which circumvents negative effects as discussed with glutaraldehyde. The reaction is enforced by *N*-hydroxysuccinimide (NHS) which reacts with the O-acylurea to become activated NHS esters. These represent activated carboxylic groups whose probability of reaction with amine groups increases. Sheep skin samples treated with NHS combined with EDC showed an increase of T_D_ by 10 K and one-third more transformed NH-groups compared to samples without this activation [248].

Petite et al. [419] activated carboxylic groups into acylazides by methylation, transfer with hydrazine and finally reaction with nitrite in aqueous saline solution. The activated carboxylic groups react with ε-amino groups to form isopeptides. But especially the esterification as first step lasted 7 days.

#### Epoxides

Bifunctional epoxides such as 1,4-butanediol diglycidyl ether (BDDGE) or ethylene glycol diglycidyl ether (EGDGE) crosslink ε-amino groups of different collagen molecules at neutral or alkaline pH [420, 421]. Kinetic investigations showed that basic and acidic catalysis, respectively is possible, but under alkaline conditions the reaction is faster and leads to stiffer tissue. Sung et al. [420] achieved T_D_ of 78 °C in porcine tendons with 4% EGDGE solution at pH 10.4 after 24 h reaction time. Lower temperatures, pH and concentration of crosslinker caused lower final T_D_.

Zeeman et al. [421] treated sheep skin collagen with BDDGE over 7 days at ambient temperatures in aqueous buffers and achieved crosslinking of carboxylic groups under acidic conditions. Subsequently, they converted free amine groups with NHS/EDC to achieve crosslinked material with T_D_ of 80 °C with high stability against enzymatic degradation.

#### Nature-derived crosslinkers

Natural crosslinkers had been used to manufacture leather for a long time. In the past, they were divided into hydrolysable tannins, which are esters from gallic acid and glucose moieties, and condensed tannins, which are polymers of catechol units. Both groups comprise typical tanning agents which were extracted from leaves, wood, galls, bark or other plant parts (for survey see [194]). These are not common to stabilize collagen for biomedical applications, though many have long been used as pharmaceutical [422–424].

Two further plant-based groups of reagents became increasingly interesting to stabilize biomaterials. The first group comprises quinones which react by nucleophilic addition with free amino groups of collagen. This is also the principle of the mussel glue which sticks the byssus to the substrate. Secretory glands produce DOPA that is further oxidized enzymatically to o-quinones which may then crosslink proteins by imin formation or by Michael addition, to name only two of several possible reactions [425–427]. Oxidizing enzymes (tyrosinase, laccase) were also directly used to activate the tyrosine of collagen which is found in the telopeptides. Tyrosinase-catalysed dopachinon reacts directly with other amino groups. Laccase leads to tyrosine radicals which dimerize to dityrosine [375]. Nordihydroguaretic acid (NDGA) is a dicatechol produced from the creosote bush which has been investigated to stabilize collagen. Koob et al. postulated that NDGA polymerizes to a matrix which encloses the collagen fibres rather than collagen is directly crosslinked [428, 429].

The other group of plant-based crosslinkers comprise selected compounds of iridoids and secoiridoids. These phytochemicals are produced by many plant families, and several thousand variants had been described and characterized [430–433]. Two representatives are genipin and oleuropein. Treating collagen with genipin causes a deep blue colour. The reaction mechanism is not completely clear, but ε-amino groups of collagen are consumed [434–436].

Oleuropein is a second example whose crosslinking activity has been investigated in the past. By treating collagen with this deglycosylated secoiridoid (pH 7; 0.2% solution; 25 °C; 0.5 U/mL β-glucosidase) T_D_ increases by 20 K and the number of ε-amino groups decreases. Though the exact reaction mechanism is not clear, the kinetics of the reaction and the stability of reaction products are similar to that of glutaraldehyde [194, 437, 438].

#### Physical crosslinking

Collagen can as well be crosslinked by different physical treatments. The most important are irradiation (UV, γ, β) and dehydrothermal treatment (DHT). However, each physical treatment causes at once also chain scissions and it depends on the conditions which effect occurs preferably [439, 440].

If irradiation is administered, crosslinking is observed more intensely when the samples are wet, measured as decrease of solubility, while in dry state chain scissions occur more frequently [439]. Monboisse and Borel [441] found that the presence of oxygen during γ- and β- irradiation leads to superoxide radicals (O_2_^−^) which they assumed to cause chain scissions. In absence of oxygen, hydroxyl radicals (OH·) are formed which lead to polymerization of soluble collagen. The exact reaction mechanisms are not known, neither for crosslinking nor for cleavage. From the engineering point of view it is very difficult to control which reaction will be preferred and this has also consequences regarding the sterilization methods.

Dehydrothermal treatment and drying are two phases of the same process. In tissue, when collagen is prepared as solution or as fibrils in aqueous systems, water covers the collagen molecules and shields them from converging. By drying, the molecules draw near and new bonds are formed. These comprise ionic and hydrophobic interactions, but also covalent bonds which may be formed e.g. between alanine and lysine. Dehydrothermal treatment is a forced temperature treatment (110 °C) at low humidites to prevent denaturation. The solubility in water decreases, but the stability against enzymatic degradation decreases as well. Therefore, denaturation of some parts of the collagen cannot be prevented [440, 442]. DHT is also used to harden gelatine [443].

### Drying

Many collagen-based biomaterials are marketed in dry state. But drying has a strong impact on the structure of the resulting material. The highly swollen structure of collagen preparations, whether solution-, suspension- or tissue-based, converge during water removal because of the action of capillary forces, T_D_ increases and to some extent new bonds can be formed. But drying allows to stabilize shapes of collagen materials. It can be used to generate pores of defined size and shape and also prolongs the storage life before use. Not least, most procedures for sterilizing collagen materials can only be applied when the materials are dry.

#### Convection drying

By convection drying the collagen fibres and molecules collapse completely, and the distances are reduced almost to a minimum. Only some water molecules remain bound to the collagen structure also in dry state [156, 444]. Nevertheless, the fibres conglutinate, and covalent reactions between the collagen molecules occur. Rehydration of such films allow re-swelling to some extent but often not to the initial state before drying.

Films are manufactured by convection drying. Flowable acidic collagen suspension or solution is cast in moulds and dried sometimes under vacuum at temperatures lower than the denaturation temperature until the equilibrium humidity is achieved. Usually, the convection-dried films show material humidities of 8 to 15%. Alternatively, collagen suspension can be extruded as cold mass through cooled flat dies and dried continuously without application of vacuum. All resulting compact films appear translucent.

#### Solvent and freeze drying

Solvent and freeze drying, respectively are applied to prevent the action of capillary forces and the collapse of the fibrous structure. During solvent drying, water is exchanged by soaking the material in increasing concentrations of alcohol or acetone (critical point drying). While this is an established technique to prepare histological preparations, the disadvantage of this procedure for production scale is the high consumption of solvent. These are flammable liquids which require special safety precautions when handling higher amounts. Alternatively, supercritical carbondioxide (CO_2_^sc^) had been used, but CO_2_^sc^ has only a low miscibility with water, and a high throughput of CO_2_^sc^ is necessary (own unpublished results).

Freeze drying as well allows to reduce capillary forces. It is the common technology to remove water from aqueous collagen preparations and to generate a porous structure. The collagen is frozen at adequate temperature gradients between − 15 and − 80 °C, and the capillary forces are suppressed by the formation of ice crystals. By sublimation of the ice under vacuum the water is removed. The final porous structure relates to the structure of the ice crystals in frozen state. This technique makes it possible to manufacture pores of a defined size. The application of steep cooling gradients up to low temperatures leads to small pores. Bigger pores are achieved by flat cooling gradients [445–448]. Aligned pores and tubelike channels of small size can be achieved by polar temperature gradients [263, 448, 449]. Freeze drying is used to manufacture sponges as flat or cylindrical applications for hemostypic applications in wound treatment and dentistry.

To manufacture porous collagen-gelatine layers without solvent or freeze drying, a gelatine solution was whisked to a foam, and fibrous collagen suspension was cautiously mixed with the gelatine foam in equal parts, applied to a supporting substrate, and the gelatine was solidified by cooling. This technique made it possible to manufacture porous structures fast and continuously [221].

### Sterilization

Each collagen material which is used in clinics or in cell culture has to be free of all forms of alife or infectious components. The used decontamination technology depends on the preparation steps and on the possible contamination in advance. If the sterilization procedure is not applied to the finally packed material at the end of the process, the subsequent steps following the sterilization procedure have to be performed under aseptic conditions.

#### Filtration

Filtration can only be used for liquid or gaseous materials which can be pressed through microfilters. Acidic collagen solutions are filtrated at low concentrations (0.1–0.2%) through 0.45 or 0.2 µ filters. This process is gentle but time-consuming, and much liquid has to be removed by drying if the final product will be applied in dry state. Then, pH is adjusted to neutral, the precipitate is centrifugated aseptically and freeze-dried [210].

#### Ethanole

Solid laboratory samples are often treated with water–ethanol mixtures (30:70%) prior to cell culture in vitro to decontaminate the material. However, it is not a sterilization technique accepted by authorities. A clearence of bacterial and fungal spores and endotoxines cannot be achieved by this technique, and other methods have to be applied when collagen biomaterials are aimed to be marketed.

#### Gamma irradiation and electron beam

The effects of γ-irradiation on living organisms has already been investigated in the beginning of the 1960s. Currently, γ-irradiation is the most common method beside ethylene oxide (EO) to sterilize dry commercial medical products from biological tissues. According to governmental standards worldwide 25 kGy have to be applied to the materials, but depending on the bioburden in some cases 15 kGy sometimes applied in several fractions are allowed as well. The radiation directly affects the DNA of living organisms. Furthermore, radicals are formed which also affect other polymeric structures such as proteins, lipids as well as endotoxins. Persistent states such as spores are less sensitive than organisms with a higher metabolism. Prions are almost not affected [450–453].

γ-Irradiation also affects the collagen structure. The observed effects are markedly influenced by the humidity of the material. At higher humidity, collagen is crosslinked. Solubility and susceptibility to enzymes often decrease. In contrast, dry collagen often loses its higher structure and is cleaved in the main chain. Furthermore, at high doses the triple helices are denatured and amide nitrogen is liberated [439, 454–457].

More recently, electron beam sterilization has been investigated as an alternative to gamma irradiation. The comparison between both methods showed no remarkable difference, but this technique is not as common as γ-irradiation [451, 458, 459].

Irradiation plants are expensive to operate because of the high safety standards. Therefore, often external suppliers perform γ-irradiation. For the customer, this leads to less controllable conditions if the material to be sterilized is e.g. sensitive to local overdosing. Fractionated radiation is possible but more expensive.

#### Ethyleneoxide (EO)

Ethyleneoxide gas, 100% or mixed with nitrogen or CO_2,_ is used as gaseous chemical sterilant at relative humidity between 50 and 80% at slightly elevated temperatures between 33 up to 45 °C for up to 90 h. EO diffuses easily through pores of the packaging (Tyvec^®^) as well as inside of collagen materials as sponges or fibrous structures. The sterilization of wet materials is not possible, however. EO is toxic, cancerogenic, mutagenic and explosive. Therefore, it is applied in closed chambers with extensive safety installations to dose and detoxify the gas. For safety reasons, it is crucial to flush the treated materials intensely with dry warm air and to evaporate any toxic remnant [460, 461].

EO is an alkylation agent. Only low or no effects on the structure or the mechanical stability of collagen materials have been described yet [462, 463]. However, effects on the side chains of collagen are still discussed controversially. Lys and Hyl groups have been observed by some authors to be modified, which led to a decreased degradation rate by collagenase [4, 462]. Others did not observe any action on primary amino groups [464].

#### Peroxoacetic acid (PAA)

PAA is used in combination with ethanole to sterilize tissue allografts e.g. skin, cartilage and ligaments. The materials are treated in wet state. The sterilant has to be completely exchanged by sterile PBS or saline solution, and this exchange has to be performed under sterile conditions. The method is permitted by the authorities to sterilize implants, though it is not an end product sterilization [451, 465, 466]. Therefore, it is required to remove all remnants of PAA by aseptic washing steps.

In collagen, available methionin is oxidized, but other amino acids seem not to be affected (own results, unpublished). Physical damage is low and other chemical properties are only slightly influenced. The efficiency of sterilization is similar to γ-treatment [451].

#### Gas plasma treatment

Gas plasma alone cannot be used to sterilize collagen materials especially sponges. The sterilizing effect is achieved by UV radiation, activated gas molecules and radicals, but the penetration depth of these species is low. Therefore, this process is only suitable to sterilize surfaces and had been established for many applications except biomaterials [467–470].

Better results are achieved when gas plasma is applied in combination with hydrogenperoxide vapour (Sterrad^®^), a recent method which has been investigated to sterilize especially medical devices like endoscopes. The effect on biomaterials has not been comprehensively investigated up to now and only one study proved the sterilization ability on collagen sponges. The sponges have not been characterized intensely. Sterilization in this layout is achieved by vaporized hydrogen peroxide which is applied in lower pressure. This leads to intense penetration in tubes but also pores. The plasma is discharged after application of vacuum. It is especially used to detoxify the hydrogen peroxide molecules in the gas phase rather than to achieve sterilization [471].

#### Supercritical carbon dioxide (CO_2_^sc^) with additives

Hydrogen peroxide and peraceteic acid (PAA) have been shown to effectively sterilize collagen materials. Therefore, freeze-dried materials are successfully sterilized by application of small amounts of hydrogen peroxide or PAA which are solved in CO_2_^sc^. The dry materials are packed in Tyvec^®^ bags which are permeable for hydrogen peroxide and CO_2_^sc^. To achieve sufficient inactivation of bacteria and spores, the pressure has to be applied in cycles and only low amounts of additive are necessary [472–477].

The technique is especially useful for collagen materials which have already been treated with oxidizing agents e.g. sponges, hernia implants or foils, because the preparation technologies often contain decontamination steps which use hydrogen peroxide [478, 479].

## Conclusion

Collagen-based medical products, which are offered on the market, are purified and prepared by routine technologies. They are applied successfully in reconstructive surgery, and the range of possible applications is further increasing. By variation of the preparation and processing techniques, collagen allows to be manufactured in many different materials whose properties can be adjusted in a broad range.

Though processing and the resulting materials had been investigated for decades, materials science and the engineering of production variants has not been maxed out yet. One reason is that much knowledge is based on tradition but another that the collagen structure and materials behaviour depends on as many surrounding conditions as humidity, kinds and concentrations of buffers, the stability of the structure of all levels including crosslinking. Not least, low amounts of additives influence the structural, chemical, physical and biological materials properties.

Yet, there are neither mathematical models which allow to predict properties of processed collagen materials nor models that consequently simulate processing and the effects of different processing steps on materials properties. Therefore, the highest potential for new developments is expected with regard to reduction of processing times to achieve sufficient purities and selected predictable materials properties in combination with sophisticated automated systems for processing and analytics.

The shape of collagen materials is currently limited to simple structures such as films, gels, powders, sponges or the saved original tissue structure of flat materials such as pericardium, SIS or dermal splits. Surgeons have to prepare more complex shapes themselves, and the recipient tissue surrounding the implant has to revascularize and replace the implanted tissue.

In recent years, decelluarization of whole organs and organ parts became more or less a routine technique which allows to prepare complex structures of the ECM by saving the original structure including the vessel trees. This complexity promised to be an advantage but a challenge remains the recellularization of such architectures, not to mention the expected requirements of purity and quality control if such materials are aimed to be accepted by the authorities.

Therefore, the field between complex decellularized organs and the marketed simple shapes such as films, sponges and powders is open for new emerging manufacturing techniques based on purified intermediates. The required standard technologies for the purification are already available to achieve cytocompatible intermediates, but there are potentials for automatization to speed up and optimize the processes.

Complex structures can be manufactured by complex combined technologies comprising different drying technologies, sterilization steps which take over further functions such as the preparation of porous structures or crosslinking in combination with casting steps such as spinning, moulding or additive manufacturing techniques. Finally, the use of further ECM derived components such as different collagen types, laminin, fibronectin, elastin or collagen-like peptides even more have the potential to manufacture complex materials but based on the knowledge how the aimed materials behave in humid biological environments. The structures can further be combined with living cells or used directly in clinics. This means that on the one hand the tool box is filled up with new emerging processing technologies and with standardized intermediates, on the other hand the combinations become more sophisticated. Automated production of complex materials is only at the beginning, however.

The major challenge will be to achieve sufficient mechanical stability and vascularization of these hybrid materials consisting of many structural elements. By now, hybrids of coarse structures can be easily manufactured and will be improved more and more in the near future because of the knowledge and supply of purified ECM based materials and more sophisticated manufacturing and shaping technologies. However, it seems more suitable not to manufacture highly complex structures such as vascular trees by engineering techniques, but to allow the biological systems to reorganize themselves which means that cells in vitro or the recipient in vivo build capillaries, nerves or metabolic structures. The biological systems have the potential to assemble simple molecules and structures to complicated architectures on the microscopic level which follow the complex physiological demands and the requirements of the individual. The use of collagen as material for additive manufacturing is only at the beginning and collagen-based peptides and the combination with other ECM derived polymers promise challenging developments.
